# E-Flux2 and SPOT: Validated Methods for Inferring Intracellular Metabolic Flux Distributions from Transcriptomic Data

**DOI:** 10.1371/journal.pone.0157101

**Published:** 2016-06-21

**Authors:** Min Kyung Kim, Anatoliy Lane, James J. Kelley, Desmond S. Lun

**Affiliations:** 1 Center for Computational and Integrative Biology, Rutgers University, Camden, New Jersey, United States of America; 2 Department of Computer Science, Rutgers University, Camden, New Jersey, United States of America; 3 Department of Plant Biology and Pathology, Rutgers University, New Brunswick, Camden, New Jersey, United States of America; 4 School of Information Technology and Mathematical Sciences, University of South Australia, Mawson Lakes, South Australia, Australia; Tata Institute of Fundamental Research, INDIA

## Abstract

**Background:**

Several methods have been developed to predict system-wide and condition-specific intracellular metabolic fluxes by integrating transcriptomic data with genome-scale metabolic models. While powerful in many settings, existing methods have several shortcomings, and it is unclear which method has the best accuracy in general because of limited validation against experimentally measured intracellular fluxes.

**Results:**

We present a general optimization strategy for inferring intracellular metabolic flux distributions from transcriptomic data coupled with genome-scale metabolic reconstructions. It consists of two different template models called DC (determined carbon source model) and AC (all possible carbon sources model) and two different new methods called E-Flux2 (E-Flux method combined with minimization of *l*^*2*^ norm) and SPOT (Simplified Pearson cOrrelation with Transcriptomic data), which can be chosen and combined depending on the availability of knowledge on carbon source or objective function. This enables us to simulate a broad range of experimental conditions. We examined *E*. *coli* and *S*. *cerevisiae* as representative prokaryotic and eukaryotic microorganisms respectively. The predictive accuracy of our algorithm was validated by calculating the uncentered Pearson correlation between predicted fluxes and measured fluxes. To this end, we compiled 20 experimental conditions (11 in *E*. *coli* and 9 in *S*. *cerevisiae*), of transcriptome measurements coupled with corresponding central carbon metabolism intracellular flux measurements determined by ^13^C metabolic flux analysis (^13^C-MFA), which is the largest dataset assembled to date for the purpose of validating inference methods for predicting intracellular fluxes. In both organisms, our method achieves an average correlation coefficient ranging from 0.59 to 0.87, outperforming a representative sample of competing methods. Easy-to-use implementations of E-Flux2 and SPOT are available as part of the open-source package MOST (http://most.ccib.rutgers.edu/).

**Conclusion:**

Our method represents a significant advance over existing methods for inferring intracellular metabolic flux from transcriptomic data. It not only achieves higher accuracy, but it also combines into a single method a number of other desirable characteristics including applicability to a wide range of experimental conditions, production of a unique solution, fast running time, and the availability of a user-friendly implementation.

## Introduction

Intracellular metabolic reactions provide a cell with biochemical building blocks, energy, and a suitable thermodynamic environment. Due to the large connectivity of metabolic networks via metabolites each of which participates in multiple metabolic reactions, determination of system-level changes in intracellular metabolic fluxes is important for understanding the fundamental mechanisms of cellular metabolic responses to environmental or genetic perturbations [1].

^13^C metabolic flux analysis (^13^C-MFA) is available for characterizing intracellular fluxes experimentally, and is the most widely accepted approach to quantify fluxes in central carbon metabolism [2]. In this approach, cells are grown on ^13^C-labeled substrates until the cells are at both metabolic steady state (i.e. when concentrations of metabolites are constant over time) and isotopic steady state (i.e. when isotopomer fractions remain stable over time). Then the level of ^13^C enrichment in cellular metabolites is measured by mass spectrometry (MS) or nuclear magnetic resonance (NMR). Finally, the intracellular flux distribution is reconstituted from the ^13^C enrichment patterns [2–6]. Though ^13^C-MFA can determine intracellular fluxes, extensive instrumentation and specialized expertise is required to carry out the experiment and the number of fluxes that can be accurately and uniquely determined is limited [7]. Moreover, in some situations, such as autotrophic growth, ^13^C-MFA is not capable of intracellular flux determination, and isotopically nonstationary MFA (INST-MFA), an even more challenging method both experimentally and computationally, is required [8].

An alternative widely used for system-level studies of metabolism is a computational modeling method called flux balance analysis (FBA). FBA defines the space of allowable metabolic flux distributions of a system at steady state by making use of genome-scale metabolic models [9]. Since these models are in general underdetermined, context-specific and physiologically meaningful flux solutions need to be narrowed down from the innumerable distributions by imposing additional constraints on the system and by optimizing certain objective functions [10].

Advances in omics technologies have enabled quantitative monitoring of the abundance of biological molecules at various levels in a high-throughput manner [11]. In the absence of complete information on regulatory rules, omics data can be integrated with genome-scale metabolic models to improve their predictive power through defining flux bounds, objective functions, or both [12]. For this purpose, transcriptomic data compares favorably to other omics platforms in that it is a platform where a complete quantitative snapshot of all genome-wide molecular species is possible [13]. In addition, RNA amount changes can be precisely measured in a highly automated process at low cost compared to the amount of data gathered [14]. Thus, integration of transcriptomics data in genome-scale metabolic models potentially enables the determination of context-specific system-wide metabolic fluxes through a relatively simple and low-cost omics technology.

There have been previous studies to integrate transcriptomic data with genome-scale metabolic models, which are well covered in recent reviews [6,11,15–20]. While powerful, existing methods have several shortcomings:

1. Some of them require multiple sets of input data for a single analysis [21,22], which is often undesirable.
2. Some methods require a user-defined threshold to define “high” or “low” expression states [23–26], which leads to subjective results since the specific threshold above which the level of gene expression indicates physiological activeness of corresponding reactions must be arbitrarily chosen and may vary across genes, conditions, or organisms.
3. Several methods require *a priori* assumption of an appropriate objective function such as biomass production rate (i.e. the growth rate) [21,24,27,28]. The biomass flux is the most widely used objective function for FBA optimization problems [29]. Although the assumption of biomass flux maximization in FBA has successfully predicted metabolic behavior, especially of fast-growing microorganisms [30], we need a method which can be universally applied to a variety of organisms in cases where knowledge of the biological objective function is uncertain, such as microorganisms with variable biomass composition, pathogens in dormancy or in latent phase, or cells of a multi-cellular organism [31].
4. Several methods produce non-unique solutions. That is, they produce a solution out of a space of possibilities, all of which are in theory possible. If all solutions in the space of possibilities were equally good in terms of their ability to predict fluxes, this would be acceptable, but in general, there is a range of possibilities for predictive accuracy. A single solution that is arbitrarily chosen is difficult to reproduce and is typically dependent on the software or hardware used for the analysis [32]. Thus, if a method has non-unique solutions, a deterministic method to pick one of the good solutions (i.e. one of the ones with high predictive accuracy) is desirable.
5. Lastly, previous studies have generally focused on conditions where the carbon source of the system and its uptake rate are known. While many biotechnological and laboratory processes operate on a known single carbon source, typically glucose, we would sometimes like to study microorganisms living in conditions where the carbon source is unknown, such as for *in vivo* applications [33].

Therefore, in light of various experimental and cellular conditions in practical applications, there is still a need for a method that can provide all five of the desirable features listed in Table 1. Moreover, until recently, the predictive accuracy of previous methods had not been tested [19].

**Table 1:** Summary of the desirable features of a method for predicting intracellular metabolic fluxes using transcriptomic data-integrated genomic models.

	Desirable features	Benefits
1	Requirement for only a single gene expression data as input	Simpler analysis with less effort and cost
2	Use of continuous gene expression values without using arbitrary thresholds	Acquisition of more fine-grained information by avoiding arbitrary classification of gene expression levels
3	Capability to be used when an appropriate objective function is unknown	Applications to microorganisms with variable biomass composition, pathogens in dormancy or in latent phase, or cells of a multi-cellular organism
4	Capability to produce a unique metabolic flux distribution	More reproducible analysis independent of hardwares and softwares used to solve optimization problems
5	Capability to be used when the carbon source of the system and its uptake rate is unknown	Applications to microorganisms living in intact tissues or in natural environments

Five desirable features of a new method are listed in the left side of the table, and the corresponding benefits are described in the right column.

**Fig 1:**
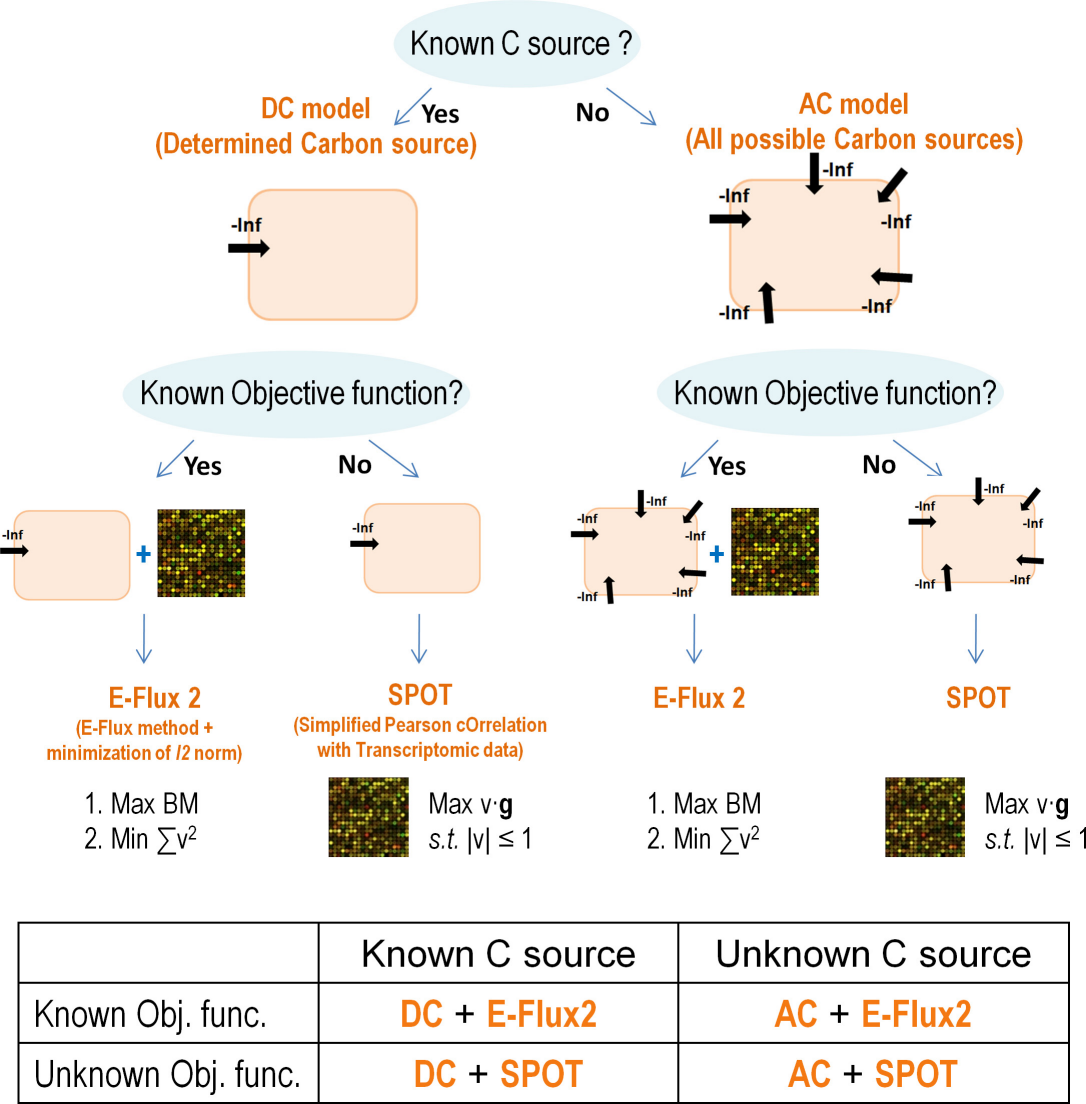
Flow chart illustrating how to choose between E-Flux2 and SPOT.

In this study, we compiled the most extensive dataset to date, consisting of 20 experimental conditions (11 in *E*. *coli* and 9 in *S*. *cerevisiae*, see Table 2 for details), of genome-wide gene expression measurements coupled with corresponding central carbon metabolism intracellular flux measurements. We used this dataset to rigorously evaluate the performance of representative methods for predicting intracellular metabolic fluxes using transcriptomic data. Based on this evaluation, we propose two new methods, E-Flux2 and SPOT, to be employed when a suitable biological objective is available and unavailable, respectively (Fig 1). The combination of the two methods provides a general strategy for predicting intracellular fluxes using transcriptomic data that satisfies all of the desirable features mentioned above. Depending on knowledge of the carbon source and availability of a suitable biological objective, this strategy achieves an average uncentered Pearson correlation of predictions against measurements over our dataset that ranges from 0.59 to 0.87, outperforming a representative selection of currently available methods.

**Table 2:** Datasets and metabolic models used for this study.

		*E*. *coli*	*S*. *cerevisiae*
Genome-scale metabolic model	For Table 3 & Figs 2 and 3	*i*JO1366 [39]	Yeast 5 [40]
	For Fig 3	*i*JO1366 [39], *i*AF1260 [41], *i*JR904 [42]	Yeast 5 [40], *i*MM904 [43], *i*ND750 [44]
Transcriptomic data & measured flux data	Dataset 1	**Ishii *et al*., 2007 [34]:** Data were measured under 8 different conditions—wild type *E*. *coli* cells cultured at a growth rate of 0.2, 0.5, and 0.7 hours^-1^, and single-gene knockout mutants (∆*pgm*, ∆*pgi*, ∆*zwf*, ∆*rpe*, ∆*gapC*); The transcriptomic data are two-color microarray data normalized using MAANOVA[45]; The number of measured fluxes used for validation—around 248 measured fluxes (31 fluxes per condition, and total 8 conditions).	**Rintala *et al*., 2009 [36] (transcriptiomic data) & Jouhten *et al*., 2008 [37] (fluxomic data):** Data were obtained from yeast cells cultured in 5 different oxygen levels (20.9, 2.8, 1.0, 0.5, and 0.0% O_2_); The transcriptomic data are single-color microarray data normalized with Robust Multichip Average normalization [46]; The number of measured fluxes used for validation—110 fluxes (22 fluxes per condition, and total 5 conditions).
	Dataset 2	**Holm *et al*., 2010 [35]:** Data were obtained from 3 different *E*. *coli* strains that are wild-type cells, NADH oxidase- overexpressing cells, and the soluble F1-ATPase- overexpressing cells; The transcriptomic data are single-color microarray data normalized using the Qspline method [47]; The number of measured fluxes used for validation—66 fluxes (22 fluxes per condition, and total 3 conditions).	**Celton *et al*., 2012 [38]:** Data were collected from yeast cells treated with 4 different concentrations of acetoin (0, 100, 200, and 300 mM); Its transcriptomic data are two-color microarray data normalized using MAANOVA [45]; The number of measured fluxes used for validation—116 fluxes (29 fluxes per condition, and total 4 conditions).
	Total	11 conditions in *E*. *coli*	9 conditions in *S*. *cerevisiae*

## Materials and Methods

A description of the whole process for our research follows below. A schematic overview of it can be found in Table A in S1 File.

### Transcriptomic data, fluxomic data, and metabolic models used for this study

The first step was to collect a dataset of transcriptomic and fluxomic measurements obtained from cells under the same conditions. The measured fluxes were obtained to compare them with the predicted fluxes. To this end, we obtained data published by Ishii *et al*.[34] and Holm *et al*. [35] for *E*. *coli*, and by Rintalta *et al*. [36,37] and Celton *et al*. [38] for *S*. *cerevisiae*, where both expression data and ^13^C flux data measured under the identical conditions can be acquired. The dataset is made up of total 20 experimental conditions (11 in *E*. *coli* and 9 in *S*. *cerevisiae*), a detailed description of which is given in Table 2.

Our analysis is mainly based on *i*JO1366 and Yeast 5 for the metabolic models of *E*. *coli* and *S*. *cerevisiae*, respectively. We also tested our methods on older models of *E*. *coli* (*i*JR904 and *i*AF1260) and those of *S*. *cerevisiae* (*i*ND750 and *i*MM904) to examine the applicability of our methods to the relatively incomplete models (Fig 3). We used the experimental datasets published in the papers listed in the lower row, each of which has both transcriptomic data and fluxomic data measured under same condition. We needed experimentally measured fluxes data to validate predictive accuracy of our methods by comparing them with the predicted fluxes. The model files are given in S1 Dataset. The transcriptomic and fluxomic data sets that were used in this study can be found in S2 Dataset.

As the metabolic models for *E*. *coli* and *S*. *cerevisiae*, we used *i*JO1366 [39] and Yeast 5 [40], respectively, in most tables and figures of this paper. As shown in Fig 3, we also tested our methods on older models of *E*. *coli* (*i*JR904 [42] and *i*AF1260 [41]) and of *S*. *cerevisiae* (*i*ND750 [44] and *i*MM904 [43]) to examine the applicability of our methods to the relatively incomplete models. The model files are given in S1 Dataset. The transcriptomic and fluxomic data sets that were used in this study can be found in S2 Dataset. The transcriptomic data and model files can be used together in MOST to reproduce our results (see S2 File).

### Creation of template metabolic models depending on carbon source information

When integrating transcriptomic data with genome-scale metabolic models, a problem of scaling can occur because the units for measuring metabolic flux and the units for measuring gene expression are not related. For instance, if the carbon uptake rate is set to 1, and the transcriptome values are all in the order of 10000, then applying such values as upper bounds will not constrain the model. To avoid this issue, we construct a template model that is independent of *a priori* information on cellular uptake rates and ATP maintenance flux. The template model is made by setting the flux bounds either to zero or to positive or negative infinity while maintaining the stoichiometric and reversibility information of the original genome-scale model:

$$a_{j}\leq v_{j}\leq b_{j}\to \bar{a_{j}}\leq v_{j}\leq \bar{b_{j}}$$ (1)

where, for all *j*,

$$\bar{a_{j}}=\left\{ \begin{array}{c} \;0,\;if\;a_{j}\geq 0,\\ -\infty ,\;if\;a_{j}<0, \end{array}\right.\;and\;\bar{b_{j}}=\left\{ \begin{array}{c} +\infty ,\;if\;b_{j}>0,\\ 0,\;if\;b_{j}\leq 0, \end{array}\right.$$

where *v* is a flux vector representing the reaction rates of the *n* reactions in the network, and *a*_*j*_ and *b*_*j*_ are the minimum and maximum reaction rates through reaction *j* defined in the original model. In this manner, we constructed two kinds of template models to simulate two different situations depending on whether we know which carbon source the cell uses. One template model, which we call 'DC (determined carbon source)', has a lower bound of negative infinity for the known carbon source uptake reaction. The other one, which we call 'AC (all possible carbon sources)', allows all carbon sources in the model to be taken up by the cell. Among all metabolites participating in the exchange reactions, the set of possible carbon sources were selected based on their chemical formula. The list of carbon sources whose uptake rate were set as negative infinity in the AC models for both microorganisms are given in S1 Table. Inorganic metabolites such as ions and water molecules were allowed to be taken up by the DC and AC models if their original genome-scale metabolic models did so. The information pertaining to each specific model we used can be found in S1 Dataset.

This step, converting original genome-scale models into DC or AC template models before integrating gene expression data, resolves the scaling problem described above. The fluxes predicted by our method have an arbitrary unit. Thus, the relative magnitude of predicted fluxes across reactions is meaningful, but their absolute magnitude is not. Any known or measured reaction rate (e.g. glucose uptake rate, ATP maintenance flux, and oxygen uptake rate that are discarded when building a DC or AC template model) can be used to normalize the predicted fluxes to an absolute reference.

### Two different optimization strategies depending on the availability of biomass objective

If information on the biomass composition of a certain organism is available and maximizing its growth rate is appropriate for prediction, our first method, called E-Flux2, is an effective way to study its metabolic behavior. Otherwise, our second method, called SPOT, can be used.

#### (1) E-Flux2

E-Flux is an extension of FBA that infers a metabolic flux distribution from transcriptomic data [27,28]. The rationale behind E-Flux is that, given a limited translational efficiency and a limited accumulation of enzyme over the time, the mRNA level can be used as an approximate upper bound on the maximum amount of metabolic enzymes, and hence as a bound on reaction rates. The standard FBA involves solving the following linear optimization problem:

$$max\;f^{\prime }v$$

$$subject\;to\;\left\{ \begin{array}{c} Sv=0\\ \bar{a_{j}}\leq v_{j}\leq \bar{b_{j}} \end{array}\right.$$ (2)

where *f* is a coefficient vector defining the organism’s objective function, *S* is the stoichiometric matrix.

The main difference between E-Flux (Eq 3 below) and the standard FBA (Eq 2 above) is that E-Flux uses *g*_*j*_, the absolute gene expression level associated with reaction *j*, for an upper bound, $b_{j}^{e}$, and sets a lower bound, $a_{j}^{e}$ = −*g*_*j*_ = ${-b}_{j}^{e}$ for reversible reactions, otherwise $a_{j}^{e}$ = 0. Here, absolute gene expression refers to any transcript abundance measurement in arbitrary units.

For one-color microarrays and RNA-seq measurements, it is relatively straightforward to determine absolute gene expression [48]. For two-color microarrays, however, it is more difficult to determine absolute gene expression because of effects such as spot size variation, and relative expression between two conditions is typically reported [49]. It is, however, possible to normalize two-color microarray data so that the gene expression levels can be compared both within an array and across arrays by estimating and removing non-biological effects, such as dye-specific, spot-specific, and array-specific effects [50,51]. For two-color microarray data (i.e. the datasets from Ishii *et al*. and Celton *et al*.), we used the MAANOVA normalization method [45] to achieve this normalization. MAANOVA uses an ANNOVA model to estimate and remove non-biological effects. We have previously used this method to successfully obtain estimates of absolute gene expression from two-color microarray measurements for E-Flux [28].

E-Flux solves the following:

$$max\;f^{\prime }v$$ (3)

$$subject\;to\;\left\{ \begin{array}{c} Sv=0\\ a_{j}^{e}\leq v_{j}\leq b_{j}^{e} \end{array}\right.$$

where, for all *j*,

$$a_{j}^{e}=\left\{ \begin{array}{c} {-g}_{j},\;if\;\bar{a_{j}}<0,\\ \;0,\;if\;\bar{a_{j}}\geq 0, \end{array}\right.\;and\;b_{j}^{e}=\left\{ \begin{array}{c} g_{j},\;if\;\bar{b_{j}}>0,\\ 0,\;if\;\bar{b_{j}}\leq 0. \end{array}\right.$$

Gene expression data were mapped to corresponding reactions in the network based on gene-protein-reaction (GPR) associations. For example, in the case where an enzyme complex consisting of subunits encoded by multiple genes mediates a certain metabolic reaction, we put the minimum value of the expression level of the associated genes on $b_{j}^{e}$ because the minimum concentration of the components determines the maximum concentration of the complete enzyme complex (See Fig A-(a) in S1 File). If a reaction is catalyzed by isozymes, we took the sum of the expression values of the associated genes for $b_{j}^{e}$ since the total capacity of the reaction is given by the sum of the capacities of its isozymes (Fig A-(b) in S1 File). If either the gene expression or GPR association relationship is unavailable for a certain reaction, then the values of $a_{j}^{e}$ and $b_{j}^{e}$ of that reaction were kept as they were defined in the template model (0 or positive or negative infinity, see Eq 1 above) so as not to constrain the model unnecessarily.

A problem of E-Flux is that the outcome solution is not unique, making it difficult to clearly identify predicted metabolic responses. Bonarius *et al*. [52] used minimization of the Euclidean norm as an objective function to find a unique metabolic flux distribution in hybridoma cells. The Euclidean norm of a vector *x*, also called the *l*^2^ norm, is given by:

$${\left|x\right|}_{2}=|x|=\sqrt{{x_{1}}^{2}+{x_{2}}^{2}+\ldots +{x_{n}}^{2}}\;for\;x=\left[\begin{array}{c} x_{1}\\ x_{2}\\ \begin{array}{c} \vdots \\ x_{n} \end{array} \end{array}\right]$$ (4)

whose intuitive geometric meaning is the length of a vector *x* on an *n*-dimensional Euclidean space R^n^. Thus, as stated by Bonarius *et al*., the constraint of minimizing the Euclidean norm corresponds to the strategy of a cell to minimize the length of the metabolic flux vector to channel metabolites as efficiently as possible. We additionally applied this theoretical constraint after maximizing the biomass flux to find a unique metabolic flux distribution satisfying both optimal biomass flux and the flux minimizing its Euclidean norm. So, the first method that we propose, which we call "E-Flux2" (meaning E-Flux method combined with minimization of *l*^2^ norm), consists of two steps of optimization, which can be chosen when a suitable objective function is known:

Step 1. E-Flux

$$z^{*}=max\;f\prime v$$ (5)

$$subject\;to\;\left\{ \begin{array}{c} Sv=0\\ a_{j}^{e}\leq v_{j}\leq b_{j}^{e} \end{array}\right.$$

↓

Step 2. Minimization of *l*^2^ norm $min\;\sum_{j=1}^{n}{v_{j}}^{2}$

$$subject\;to\;\left\{ \begin{array}{c} Sv=0\\ a_{j}^{e}\leq v_{j}\leq b_{j}^{e}\\ f\prime v=z^{*} \end{array}\right.$$

After calculating the optimal biomass flux, denoted as z* here, this method finds a unique metabolic flux distribution by minimizing the Euclidean norm of the flux vector. The square root function was ignored since removing the square root does not change the solution. Since the objective function, the Euclidean norm squared, is strictly convex, and all equality and inequality constraints are linear, which is convex, the solution of E-Flux2 is unique because the optimal solution to the problem of minimizing a strictly convex function over a convex set is unique [53]. The output vector calculated by E-Flux2 can be biologically interpreted as a metabolic flux distribution that allows the cell to achieve maximum growth rate in an energy efficient way. The idea underlying E-Flux2 is similar to parsimonious FBA (pFBA) in which FBA is followed by minimization of the *l*^*1*^ norm (or Manhattan norm) [54]. pFBA does not, however, necessarily produce a unique solution since the objective function, the *l*^*1*^ norm, is not strictly convex.

Though not largely different from E-Flux, E-Flux2 overcomes a major shortcoming of E-Flux, namely, that it does not yield a unique solution. Among the space of solutions that E-Flux provides, E-Flux2 provides a method to select one solution in a manner that is intuitive and yields high correlation to measured fluxes (see Results and Discussion).

#### (2) SPOT

If a suitable objective such as the biomass flux is unknown, we can use a second method which is to maximize correlation between a flux vector, *v*, and its corresponding gene expression data, *g*. The assumption behind this strategy is that enzymatic transcript concentrations and metabolic fluxes can be related to each other, albeit in a complex manner, since the existence of a transcript is necessary for the presence or activity of its corresponding enzyme [55]. To calculate the correlation, we used the uncentered Pearson product-moment correlation which is a popular measure of the linear correlation between two variables, resulting in the following optimization problem:

$$\max\;\frac{v∙g}{\|v\|\|g\|}=\frac{\sum_{j=1}^{n}v_{j}g_{j}}{\|v\|\|g\|}$$ (6)

$$subject\;to\;\left\{ \begin{array}{c} Sv=0\\ \bar{a_{j}}\leq v_{j}\leq \bar{b_{j}} \end{array}\right.$$

where,forallj=1,…,n,

$$g_{j}=\left\{ \begin{array}{c} g_{j},\;if\;g_{j}\;is\;available\;and\;\bar{a_{j}}\geq 0,\\ {-g}_{j},\;if\;g_{j}\;is\;available\;and\;\bar{b_{j}}\leq 0,\\ 0,\;otherwise\; \end{array}\right.$$

we will consider a problem with modified upper and lower bounds that are 0 or ± infinity as described in Eq 1. If the network contains reversible reactions, the objective function of problem (6) is potentially problematic because the directions of reversible reactions (signs of their fluxes) are unknown, while gene expression is always positive as shown in Fig B-(a) in S1 File. We therefore decomposed each reversible reaction *j* into two positive irreversible reactions, the forward reaction, $v_{j}^{f}$, and the backward reaction, $v_{j}^{b}$, where $v_{j}=v_{j}^{f}-v_{j}^{b}$, and $v_{j}^{f},\;v_{j}^{b}\geq 0$ (Fig B-(b) in S1 File). Let us assume without loss of generality that reactions 1,…,*n* − *r* are irreversible, while reactions *n* – *r* + 1,…,*n* are reversible, and that all irreversible reactions are defined in the forward reactions (i.e. their fluxes are non-negative). Then, instead of problem (6), we solve:

$$\max\;\frac{\bar{v}∙\bar{g}}{\|\bar{v}\|\|\bar{g}\|}=\frac{\sum_{k=1}^{n-r}v_{k}g_{k}+\sum_{k=n-r+1}^{n}v_{k}^{f}g_{k}+\sum_{k=n-r+1}^{n}v_{k}^{b}g_{k}}{\|\bar{v}\|\|\bar{g}\|}$$ (7)

$$subject\;to\;\left\{ \begin{array}{c} \bar{S}\bar{v}=0\\ v\geq 0\\ v^{f}\geq 0\\ v^{b}\geq 0 \end{array}\right.$$

where $\bar{v}={\left[v^{irr}{\;v}^{f}\;v^{b}\right]}^{T}={\left[v_{1}\;\cdots \;v_{n-r}\;v_{n-r+1}^{f}\;\cdots \;v_{n}^{f}\;v_{n-r+1}^{b}\;\cdots \;v_{n}^{b}\right]}^{T}$, $\bar{g}={\left[g^{irr}{\;g}^{rev}\;g^{rev}\right]}^{T}={\left[g_{1}\;\cdots \;g_{n-r}\;g_{n-r+1}\;\cdots \;g_{n}\;g_{n-r+1}\cdots g_{n}\right]}^{T}$, $\bar{S}=[S^{irr}\;S^{rev}\;{-S}^{rev}]$, *S*^*irr*^ is the submatrix consisting of the first *n* – *r* columns of *S*, and *S*^*rev*^ is the submatrix consisting of columns *n* – *r* + 1 to *n* of *S*. Solving this optimization problem is computationally inefficient since the form of the objective function is nonlinear. However, this problem can be converted to an equivalent semi-definite programming problem (8):

$$\max\;\bar{v}∙\bar{g}$$ (8)

$$subject\;to\;\left\{ \begin{array}{c} \bar{S}\bar{v}=0\\ 0\leq \bar{v}\\ {\left\|\bar{v}\right\|}^{2}\leq 1 \end{array}\right.$$

This is the second method we propose, which we call "SPOT" (Simplified Pearson cOrrelation with Transcriptomic data). SPOT can be used when biomass flux is not an appropriate optimization objective. The conversion of optimization problem (7) to SPOT (8) is based on a few steps of justification.

First, the maximum Pearson product-moment correlation is not dependent on the length of the flux vector, $\bar{v}$ (see Fig B-(c) and Supplementary Methods in S1 File). Thus, the norm of $\bar{v}$ can be ignored in the objective function. Since the norm of $\bar{g}$ is a constant that only affects the objective value, not the optimal flux distribution, it was also removed in the objective function. Lastly, to avoid the situation where the maximum value of $\bar{v}∙\bar{g}$ goes to infinity, the norm of $\bar{v}$ was constrained to an arbitrary number, in this case, 1.

The optimization problem described in Eq 7 can be simplified to SPOT only if the maximum correlation is independent of the length of the flux vector, $\bar{v}$. This is true provided the allowable flux solution space includes the origin, which is indeed the case. Fig B-(d) in S1 File explains this geometrically. The solution of (8) is unique (see Supplementary Methods in S1 File for the proof).

Transcriptomic data are used to constrain fluxes in the model for E-Flux2, and they are used to define the objective function for SPOT. The process of making a choice between E-Flux2 and SPOT is described in the flow chart in Fig 1.

If we know the cell's carbon source, we use the DC (determined carbon source) template model which has a negative infinity value on the lower bound of the known carbon source uptake reaction. Otherwise, we use the AC (all possible carbon sources) model which allows all carbon sources in the model to be taken up by the cell. If the biomass composition of the cell is known and the maximization of biomass flux is a suitable objective function, E-Flux2 (E-Flux method and minimization of *l*^*2*^norm) can be used. Otherwise, we can use SPOT (Simplified Pearson cOrrelation with Transcriptomic data).

### Validation of the predictive accuracy of the algorithm using the measured fluxes

The predictive accuracy of our algorithm was validated by calculating the uncentered Pearson product-moment correlation between *in silico* fluxes and corresponding ^13^C-determined *in vivo* intracellular fluxes, that is

$$\frac{v_{p}∙v_{m}}{\left\|v_{p}\|\|v_{m}\right\|}$$ (9)

where *v*_*p*_ and *v*_*m*_ are the predicted and measured vectors of intracellular fluxes, respectively, and ‖∙‖ denotes the *l*^2^ norm. The uncentered Pearson correlation is a good metric of the performance of flux inference methods because these methods allow determination of fluxes only within an unknown scale factor. A value of the correlation coefficient close to +1 or -1 indicates a strong positive or negative linear relationship between *v*_*p*_ and *v*_*m*_, respectively. A value of 0 indicates no linear relationship [56].

We found that some of the measured fluxes are not directly matched with predicted fluxes of the model in a 1-to-1 relationship since the reactions described in the model are more detailed. Like the GPR association relationships that were used to match genes with corresponding reactions, we identified OR or AND relationships between predicted fluxes (Fig A-(c) and A-(d) in S1 File). If a measured reaction corresponds to the set of consecutive reactions in the model that are linked with intermediate metabolites (AND relationship, Fig A-(c) in S1 File), then the minimum flux value—the slowest reaction rate—among those predicted fluxes was used to calculate correlation with the corresponding measured flux since the rate of a reaction with several steps is determined by the slowest step, which is known as the rate-limiting step in chemical kinetics [57]. If a measured flux corresponds to multiple identical reactions (OR relationship, Fig A-(d) in S1 File), the sum of those predicted fluxes was used to calculate the correlation since the rate of a reaction would be faster, that is, would have greater flux value, as the number of reactions that can perform an identical chemical conversion increases.

The reactions whose measured fluxes were used to calculate the correlation for each dataset are shown in S2 Dataset. It should be noted that our validation is directly based only on these reactions and, in general, they belong to central carbon metabolic pathways. We hypothesize that our flux predictions for other reactions (e.g., reactions in secondary metabolism) are likely also to be good, given the interconnected nature of metabolism, but our data do not allow us to directly test this hypothesis. Another thing to note is that all data were gathered from cells grown on glucose. There are, therefore, significant similarities among all the measured flux distributions, and indeed, it is possible to find a single flux distribution for *E*. *coli* and a single flux distribution for *S*. *cerevisiae* that each achieve high correlations with the measured data in each organism (data not shown). Nevertheless, the dataset we have gathered is the largest and most comprehensive dataset that currently exists for validating methods of predicting intracellular fluxes from transcriptomic data. We expect that the high correlations obtained by E-Flux2 and SPOT will generalize beyond *E*. *coli* and *S*. *cerevisiae* growing on glucose, given how their underlying optimizations reflect our general understanding of the relationship between metabolic flux and gene expression, but we cannot conclude this without additional data. In particular, coupled trascriptomic and fluxomic data obtained in organisms under very different conditions (e.g., organisms growing photoautotrophically or organisms under non-growth conditions) would help significantly in establishing the generality of our method.

### Algorithm implementation of our methods

All methods in this study initially implemented in MATLAB (The Mathworks, Inc., Natick, Mass.). These were tested using MATLAB R2013b with Gurobi Optimizer 5.6 (Gurobi Optimization, Inc., Houston, Texas). SBMLToolbox was used to convert an SBML (Systems Biology Markup Language) model into a MATLAB data structure [58]. Computations were carried out on the Window 8 platform using a personal computer with an Intel Core i5 3.10 GHz processor with 8 GB of RAM. E-Flux2 and SPOT methods are also implemented in a freely downloadable software package called MOST (Metabolic Optimization and Simulation Tool) which is available at http://most.ccib.rutgers.edu/ whose source code is open to the public [59].

## Results and Discussion

### Validation of the accuracy of our predictions against measured intracellular fluxes

The Pearson correlation between the predicted and the measured intracellular fluxes was calculated to validate the predictive accuracy of our method. All correlation values used to draw figures and tables are summarized in S2 Table. The correlation values were grouped into four different cases depending on the availability of carbon source or objective function information. Biomass flux and glucose were used as the known objective function and the known carbon source in this study. The bold number in each category of the table presents the average correlation of 11 samples in *E*. *coli* and 9 samples in *S*. *cerevisiae*. The number on the right side of the plus minus sign indicates its standard deviation.

As summarized in Table 3, overall, the predicted fluxes of our method showed good correlation with the measured fluxes both in *E*. *coli* and *S*. *cerevisiae*. The result implies that our method can predict the measured intracellular fluxes best when we have knowledge of both carbon source and objective function (DC+E-Flux2, average correlation: 0.8683). Our algorithm is able to predict intracellular metabolic fluxes with a good correlation if the information on either biomass objective or carbon source is unknown as we can see in the category of DC+SPOT (average correlation: 0.8030), and AC+E-Flux2 (average correlation: 0.6733). In the case where there is no information on both carbon source and biomass objective, our AC+SPOT method allows us to predict intracellular metabolic fluxes with an average correlation of 0.5927. Although this value is weaker than those of the other three cases of our method, a Pearson correlation coefficient around 0.6 nevertheless represents moderate positive correlation [60].

**Table 3:** Validation of the accuracy of our predictions against measured intracellular fluxes.

	Known C source (glucose, in this case)					Unknown C source	
Known objective function (biomass, in this case)	Standard FBA^1)^	pFBA^1)^	FBA+min *l*^*2*^	DC+E-Flux^1)^	**Our method** (DC+E-Flux2)	AC+E-Flux^1)^	**Our method** (AC+E-Flux2)
	Known C uptake rate			Unknown C uptake rate			
	**0.7952** ± 0.2317 [0.4965, 0.8516]	**0.8337** ± 0.1800	**0.8106** ± 0.1740	**0.7829** ± 0.1307 [0.3506, 0.9223]	**0.8683** ± 0.0964	**0.4516** ± 0.2343 [0.0027, 0.8625]	**0.6733** ± 0.1349
Unknown objective function	DC+Lee *et al*.			**Our method** (DC+SPOT)		AC+Lee *et al*.	**Our method** (AC+SPOT)
	**0.5792** ± 0.3642			**0.8030** ± 0.0342		**0.1257** ± 0.1268	**0.5927** ± 0.0974

The Pearson correlation between the predicted and the measured intracellular fluxes was calculated to validate the predictive accuracy of our method. The correlation values were grouped into four different cases depending on the availability of carbon source or objective function information. The bold number in each category of the table presents the average correlation of 11 samples in *E*. *coli* and 9 samples in *S*. *cerevisiae*. The number to the right of the ± indicates its standard deviation. Since the fluxes predicted by standard FBA, pFBA and E-Flux are not unique, the output flux obtained using our specific implementation was used to calculate the average correlation. For FBA and E-Flux solutions, the minimum and the maximum correlations between predicted fluxes and the measured fluxes that each method can theoretically achieve are given within square brackets after their average correlations. The way that we calculated the possible range of correlations of each method is described in Supplementary Methods in S1 File. Note that the maximum possible correlation can be calculated only when we already have the measured flux datasets. There is no way to force each method to produce a metabolic flux distribution that achieves the best correlation with the measured fluxes. Our methods, E-Flux2 and SPOT, were developed during the process of testing various strategies for producing unique flux distributions and identifying those that achieve good correlation on average with measured fluxes.

^1)^ metabolic flux distributions produced by these methods—FBA, pFBA, E-Flux- are not unique

To see whether the good or modest correlation value between predicted and measured fluxes in each case is because of a good correlation between transcripts and measured fluxes itself, we also calculated the correlation between gene expression data and measured fluxes. When calculating the correlation with gene expression data, we used the absolute values of measured metabolic fluxes since gene expression values are always positive, while we used the signed measured fluxes when calculating the correlations with predicted fluxes. The correlation between gene expression data and the absolute values of the measured fluxes was 0.4923 (standard deviation: 0.2900), which is weaker than all of the correlations between the predicted fluxes and the measured fluxes. Although this value cannot be directly compared with correlations within the table (since they are calculated differently), this relatively poor correlation between gene expression data and the measured fluxes, given as a point of comparison, suggests that the correlation is improved by incorporating the gene expression data into a genome scale model.

### Comparison of correlation with competing methods

Using the same transcriptomic and fluxomic datasets, we compared the accuracy of our predictions with other competing methods. We chose to compare against E-Flux [27,28] and the approach of Lee *et al*. [61], which are representative of competing methods which use a single transcriptomic dataset for an analysis without thresholds. Moreover, these two methods were compared against other methods of a similar nature including GIMME [24] and iMAT [23,25], and showed better performance in predicting exometabolome fluxes [61] or in robustness analysis [19]. For the Lee *et al*. method, we used an implementation provided with the original publication.

In all four scenarios with varying availability of carbon source or biomass objective information, our method outperformed existing methods in that it showed a higher average correlation with a smaller standard deviation (Table 3). Particularly when the carbon source is known but the biological objective is unknown, the Lee et al. method gives better predictions in *E*. *coli* (average correlation: 0.8887) but worse predictions in yeast (average correlation: 0.2009) than our method (DC+SPOT) whose average correlation is 0.7960 and 0.8117 in *E*. *coli* and in yeast, respectively (S2 Table). Unlike a prokaryote model such as *i*JO1366, a eukaryote model such as Yeast 5 is compartmentalized into organelles (e.g. mitochondria, peroxisomes, lysosomes, ER, and nucleus). As can be seen in S2 Dataset, the set of measured intracellular fluxes that were used for validation includes inter-organelle transport reactions such as pyruvate transport between cytoplasm and mitochondria where the incorrect predictions of the Lee et al. method mainly occurred. Considering the importance of compartmentalization in eukaryotic metabolic models [62], our method seems to be more desirable to study more complex systems since it is less influenced by whether the model is compartmentalized or not.

We also have carried out standard FBA and parsimonious FBA (pFBA) for reference [54]. pFBA was performed using the COBRA Toolbox [63]. Since standard FBA and pFBA require *a priori* information on several specific fluxes such as sugar (e.g. glucose) uptake rate and oxygen exchange rate, these fluxes were set according to the experimental conditions described in the four papers where the transcriptomic and fluxomic data sets were obtained. Simulating anaerobic growth with Yeast 5 requires the simulated medium to be supplemented with phosphadiate and sterols and modification of the biomass definition [40]. Due to inconsistency with the experimental condition, we could not evaluate the performance of standard FBA and pFBA in the 0% oxygen condition of the Rintalta *et al*. dataset (S2 Table).

Since standard FBA and pFBA need both carbon source and objective function information, their correlations can be compared with those of E-Flux and E-Flux2 in Table 3. Our method (0.8683, SD: 0.0964) performs better than standard FBA (0.7952, SD: 0.2317) and pFBA (0.8337, SD: 0.1800) in terms of both the correlation and the standard deviation. In the previous study by Machado and Herrgård [19], pFBA has been shown to have a higher overall predictive capability over various methods that integrate gene expression data, which casts doubt on the necessity of utilizing transcriptomic data in constraint-based modeling. Our result, however, suggests that integration of gene expression data can be used to improve flux distribution predictions, particularly when the carbon and oxygen uptake rates are unknown.

Importantly, the result predicted by our method is unique. The Lee et al. method also produces a unique solution using geometric FBA [32], which identifies the center of a solution space. Since halfway between infinity and zero or between plus and negative infinity is not defined, we set lower and upper bounds of the models to either zero or ±1000 (1000 is chosen as an arbitrary, large number) to run the Lee et al. method. For standard FBA and E-flux, which do not necessarily produce a unique flux distribution, and can produce flux distributions within a set of possibilities, the possible range of correlation (from the minimum to the maximum) between the measured fluxes and the predicted fluxes was calculated, which is presented within square brackets next to the average correlation. The calculation of these ranges is described in Supplementary Methods in S1 File. pFBA also does not necessarily produce a unique flux distribution (as discussed in Methods), but calculation of the possible range of correlation is complex, and we have therefore omitted it.

In addition, we performed FBA along with the minimization of *l*^*2*^ norm (Table 3, denoted as FBA+min *l*^*2*^). It also showed good correlation with measured fluxes (0.8106, SD: 0.1740). When knowledge of uptake rates is available, the FBA+min *l*^*2*^ method is a good alternative to pFBA, since it is easier to implement and produces a unique metabolic flux distribution.

### Detailed quantitative features of the predicted fluxes

**Fig 2:**
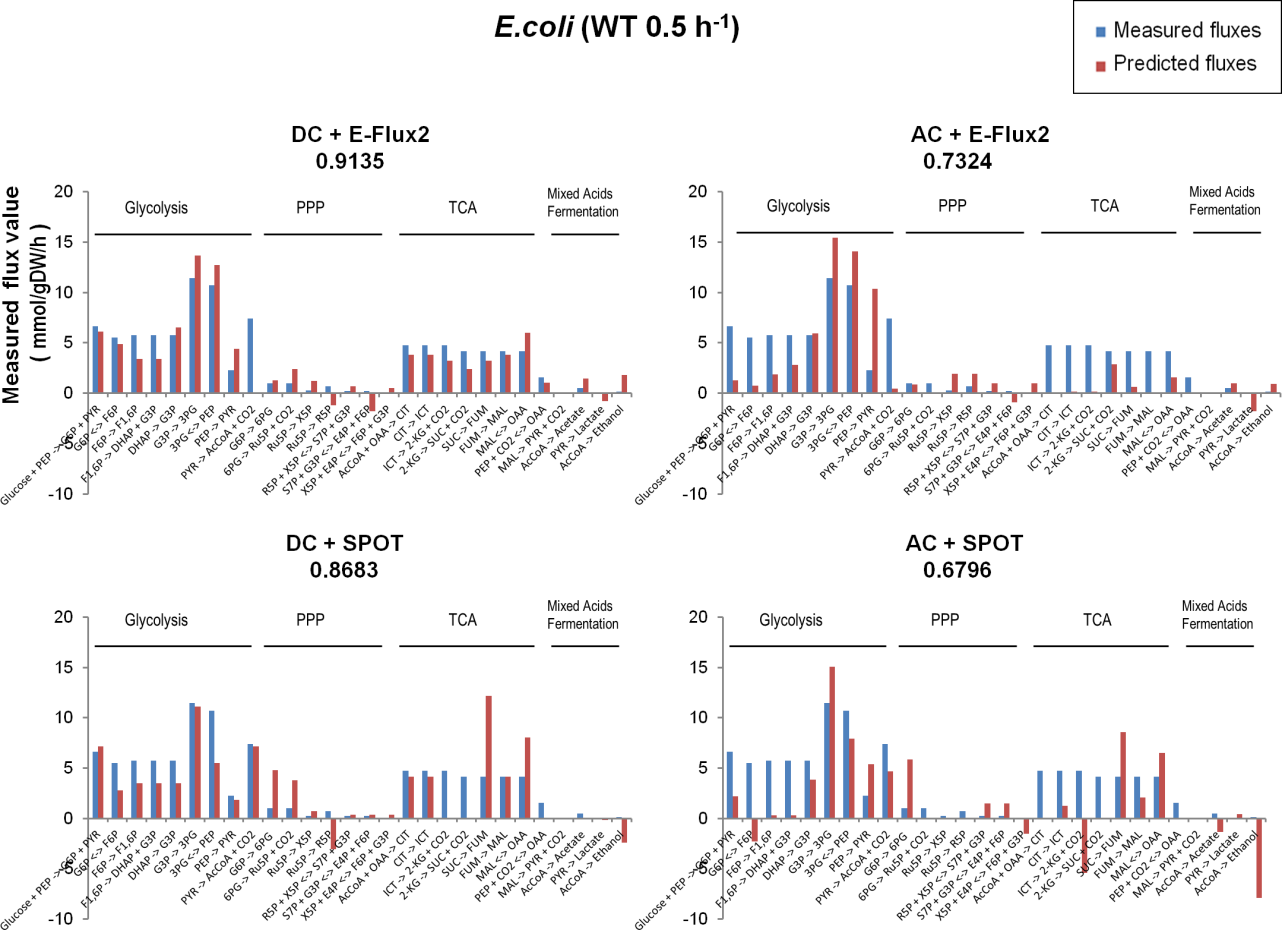
Comparison of the predicted fluxes with the measured fluxes of *E*. *coli* data (WT 0.5h^-1^ sample). The *x*-axis represents metabolic reactions used to calculate correlation between the measured (blue bars in the figure) and the predicted fluxes (red bars in the figure), and the *y*-axis indicates flux value. The scale and the units on the *y*-axis are based on those of the measured flux.

In addition to calculating correlations, we examined how the predicted and the measured metabolic flux distribution visually compare to each other. Since the predicted flux of our method has an arbitrary unit, the magnitudes of the predicted fluxes were normalized by the Euclidean norm of the measured flux vector for comparison. The results are shown in Fig 2 and Fig C in S1 File for *E*. *coli* and *S*. *cerevisiae*, respectively. The *x*-axis represents a set of metabolic reactions used to calculate correlation between the measured and the predicted fluxes, and the *y*-axis indicates flux value. The scale and the units on the y-axis are based on those of the measured flux. The reactions are grouped functionally based on the pathways in which they are participating such as glycolysis and the tricarboxylic acid cycle. As can be seen in the figure, the predicted and the measured metabolic flux distribution look similar to each other when the correlation between them is high. We see moreover that AC+SPOT predicts negative fluxes for some reactions which are supposed to be positive, which might explain one of the reasons why the method shows relatively poor correlation compared to the other three methods (DC+E-Flux2, AC+E-Flux2, and DC+SPOT). Based on this observation, possible ways to improve the correlation of AC+SPOT will be discussed in the following section.

### Test of our methods on previous models of *E*. *coli* and *S*. *cerevisiae*

**Fig 3:**
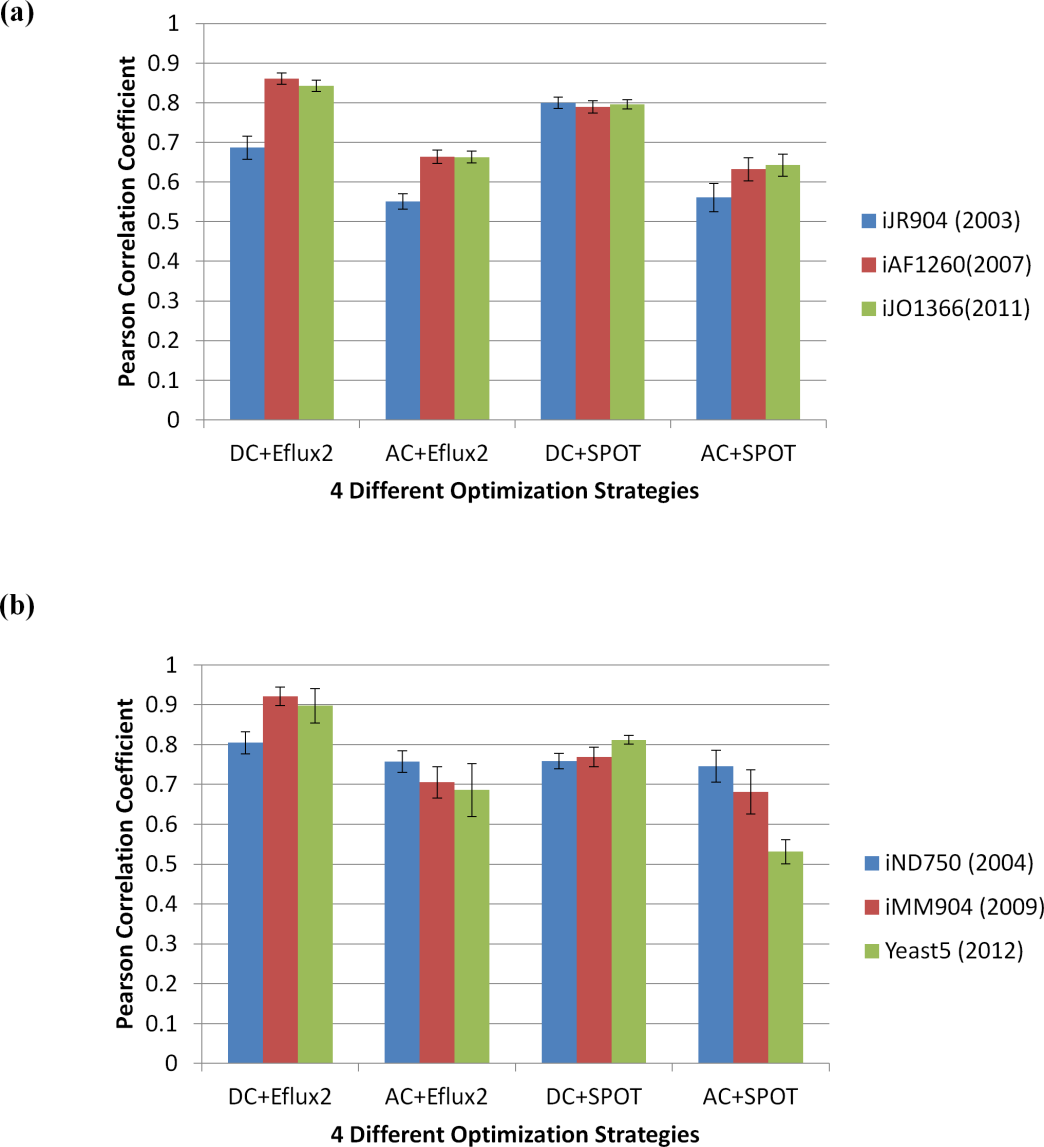
Test of our methods onto older models of *E*. *coli* and *S*. *cerevisiae*. We tested our methods on older models of *E*. *coli* (*i*JR904 and *i*AF1260) and those of *S*. *cerevisiae* (*i*ND750 and *i*MM904) to examine the applicability of our methods to the relatively incomplete models. The *x*-axis represents the four different optimization strategies and the *y*-axis identifies the average Pearson correlation coefficient between the predicted fluxes and the measured fluxes of *E*. *coli* (Fig 3a) and *S*. *cerevisiae* (Fig 3b). Error bars represent standard error of the mean (SEM).

*E*. *coli* and *S*. *cerevisiae* are two of the most intensively studied model microorganisms. On the other hand, genome-scale metabolic models of many other organisms are still incomplete. Thus, it is important to examine the applicability of our methods to relatively incomplete models before applying our methods to other organisms. One of the ways to test this is by running our methods on older models of *E*. *coli* and *S*. *cerevisiae* that are relatively incomplete. Using the same transcriptomic and fluxomic datasets, we tested our methods on older models of *E*. *coli* (*i*JR904 and *i*AF1260) and of *S*. *cerevisiae* (*i*ND750 and *i*MM904), and the results obtained are shown in Fig 3. In this figure, the *x*-axis represents the four different optimization strategies (denoted as DC+E-Flux2, AC+E-Flux2, DC+SPOT and AC+SPOT) and the *y*-axis identifies the average Pearson correlation coefficient between the predicted fluxes and the measured fluxes of *E*. *coli* (Fig 3A) and *S*. *cerevisiae* (Fig 3B). Each optimization strategy consists of a group of three bars among which the blue, red, and green bars indicate the average correlation of the oldest, middle, and newest models. Error bars represent the standard error of the mean (SEM).

In case of *E*. *coli* (Fig 3A), the two recent models (*i*AF1260 and *i*JO1366) showed better average correlation than the oldest model (*i*JR904) in most cases. We found that there is little difference in the average correlation between *i*AF1260 and *i*JO1366.

In the case of *S*. *cerevisiae* (Fig 3B), the newest model (Yeast 5) achieves a correlation that is essentially as good as or better than earlier models (*i*ND750 and *i*MM904) when the carbon source is known (DC+E-Flux2 and DC+SPOT). Unlike the *E*. *coli* case, however, the newest yeast model performs worse than the older models when carbon source of a yeast cell is unknown, especially for AC+SPOT. To understand why, we explored the hypothesis that the reason for the poor performance of Yeast5 is because of the larger number of carbon sources (see Fig E in S1 File for details). Although we could not fully identify the reason, it seems that the larger number of carbon sources has something to do with its decrease performance, but is certainly not the whole story. A different degree of interconnectivity among intracellular and exchange reactions inherent to each model or other unknown factors (e.g. thermodynamically infeasible cycles and dead-end metabolites that are unintentionally added to a newer model) may also play a role.

Except for *i*JR904 in *E*. *coli*, DC+E-Flux2 showed the highest average correlation (between 0.8 and 0.9) in both microorganisms. Thus, if we study an organism where information on both carbon source and objective function is known, applying DC+E-Flux2 is recommended.

Interestingly, DC+SPOT (known carbon source and unknown objective function) shows steady and constant average correlation between 0.7 and 0.8 in both *E*. *coli* and *S*. *cerevisiae* regardless of which model was used. The method seems to be the least influenced by incompleteness of the model. Thus, DC+SPOT is useful for predicting intracellular metabolic flux distribution in less well-studied organisms.

### Measurement of the speed of our methods

From a practical perspective, short running time is a desirable characteristic. Thus, we measured the running time of our algorithm for all 80 samples (4 optimization strategies and 20 samples per strategy) using the built-in MATLAB function, profile. The execution time for our method is illustrated in Fig D in S1 File. Regardless of which simulation strategy is used, our method, including mapping the transcriptomic data, solving the optimization problem, predicting the intracellular metabolic flux distribution and calculating the correlation with the measured fluxes, can be performed within two seconds for both microorganisms.

### Implementation of our methods in a user-friendly interface

As an ultimate representation of the cellular metabolic phenotype, metabolic fluxes provide important information to understand the functioning of cellular processes [5]. Our methods which allow to quickly and easily determine metabolic fluxes from gene expression data, thus, will be of interest to a wide audience in various biological fields. For possible users of our method especially who are not skilled in computer programming, we implemented E-Flux2 and SPOT in an intuitive user-friendly interface called MOST to make our methods viable to all researchers regardless of whether they are trained in computer science or not. MOST (Metabolic Optimization and Simulation Tool, http://most.ccib.rutgers.edu/) is an open source-based software package for constraint-based modeling [59]. It provides Excel-like editing functionality as well as supports Systems Biology Markup Language (SBML) and Comma Separated Value (CSV) files. How to run our E-Flux2 and SPOT in MOST is described in S2 File.

## Conclusions

In this study, we developed a computational method to infer intracellular fluxes from transcriptomic data using genome-scale models, which satisfies desirable features summarized in Table 1. On top of that, the predictive accuracy of our method was validated against measured intracellular fluxes, and we found it to be more accurate than existing methods.

Our method can also be easily applied to study the metabolic flux distributions of various engineered strains with little or no modification to genome-scale models since transcriptomic data themselves reflect knock-outs, knock-ins (with addition of metabolic reactions into the model that correspond to the knocked-in gene), induced-amplification or induced-repression of metabolic genes. In addition, E-Flux2 is flexible in that if there is an alternative objective to maximizing growth rate that is considered more applicable to a certain organism, then the biomass flux used in the first optimization step of E-Flux2 can be replaced with this objective function.

The multiple advantages of our method make it a useful tool for identifying fundamental mechanisms of metabolic responses and finding molecular targets for metabolic engineering. For instance, by using this tool with a set of gene expression data measured over a time course, we can determine how intracellular metabolic flux changes and where significant redirection occurs. Our method is available in a user-friendly, open source-based software package called MOST (http://most.ccib.rutgers.edu/).

## Supporting Information

S1 Dataset Genome scale metabolic models used in this study. Three *E*. *coli* models (*i*JR904 [42], *i*AF1260 [41], and *i*JO1366 [39]) and three *S*. *cerevisiae* models (*i*ND750 [44], *i*MM904 [43], and Yeast 5 [40]) used in this study are provided in Systems Biology Markup Language (SBML) format. Both DC and AC versions of each model are available.
(ZIP)

S2 Dataset Transcriptomic and fluxomic data used for this study. The transcriptomic and the fluxomic data sets that were used in this study can be found in the first four sheets of this Excel file and on the fifth sheet, respectively.
(XLS)

S3 Dataset MATLAB code that implements our method. Instructions on using it with the dataset of Ishii et al. can be found in README.doc. The source code written in Java is also available (see S2 File for details). This MATLAB code includes calculation of the Pearson correlation with measured flux data.
(ZIP)

S1 File Supplementary figures and methods. This file contains five Supplementary figures (Fig A. Schematic overview of this study, Fig B. Rationale for the SPOT method, Fig C. Comparison of the predicted fluxes with the measured fluxes of *S*. *cerevisiae* data, Fig D. Average running time of our algorithm, Fig E. Exploration of the way to improve a poor performance of Yeast 5 in AC+SPOT) and three supplementary methods (A mathematical justification for dropping the ‖*v*‖ term in the objective function of SPOT, A mathematical proof of the uniqueness of SPOT solutions, Calculation of the possible range of correlation between the measured fluxes and the predicted fluxes).
(PDF)

S2 File Tutorial on how to run E-Flux2 and SPOT in MOST. E-Flux2 and SPOT are implemented in the open source package MOST (http://most.ccib.rutgers.edu/). This document covers information on how to reproduce our results in MOST using the model files and transcriptomic data that are provided as Datasets S1 and S2.
(DOC)

S1 Table List of potential carbon source uptake reactions in *E*. *coli* and *S*. *cerevisiae* models. The lower flux bounds of these reactions were relaxed to -Inf to construct AC template models for each microorganism (i.e. to simulate the condition where knowledge of carbon source is unavailable).
(XLS)

S2 Table Detailed correlation values for tables and figures. All the Pearson correlation values between predicted fluxes of our method and measured fluxes, that are used for producing Table 3 and Fig 3, are summarized.
(XLS)

S3 Table Common exchange reactions of the possible carbon sources across three yeast models. Total 106 exchange reactions that are common across all three yeast models (*i*ND750, *i*MM904, and Yeast 5) were identified to test the hypothesis that the number of carbon source uptake reactions affects the performance of the models.
(XLS)
