# Supplementary material for: E-Flux2 and SPOT: Validated Methods for Inferring Intracellular Metabolic Flux Distributions from Transcriptomic Data
Source: PLoS One. 2016 Jun 21;11(6):e0157101. doi: 10.1371/journal.pone.0157101 (PMC4915706; doi:10.1371/journal.pone.0157101)
Supplement: S1 File — This file contains five Supplementary figures (Fig A. Schematic overview of this study, Fig B. Rationale for the SPOT method, Fig C. Comparison of the predicted fluxes with the measured fluxes of S. cerevisiae data, Fig D. Average running time of our algorithm, Fig E. Exploration of the way to improve a poor performance of Yeast 5 in AC+SPOT) and three supplementary methods (A mathematical justification for dropping the ‖v‖ term in the objective function of SPOT, A mathematical proof of the uniqueness of SPOT solutions, Calculation of the possible range of correlation between the measured fluxes and the predicted fluxes). (PDF) [file pone.0157101.s004.pdf]

Supplementary figures and methods

E-Flux2 and SPOT: Validated methods for inferring intracellular metabolic flux distributions from transcriptomic data

Min Kyung Kim, Anatoliy Lane, James J. Kelley, and Desmond S. Lun

Supplementary figure 1. Schematic overview of this study.

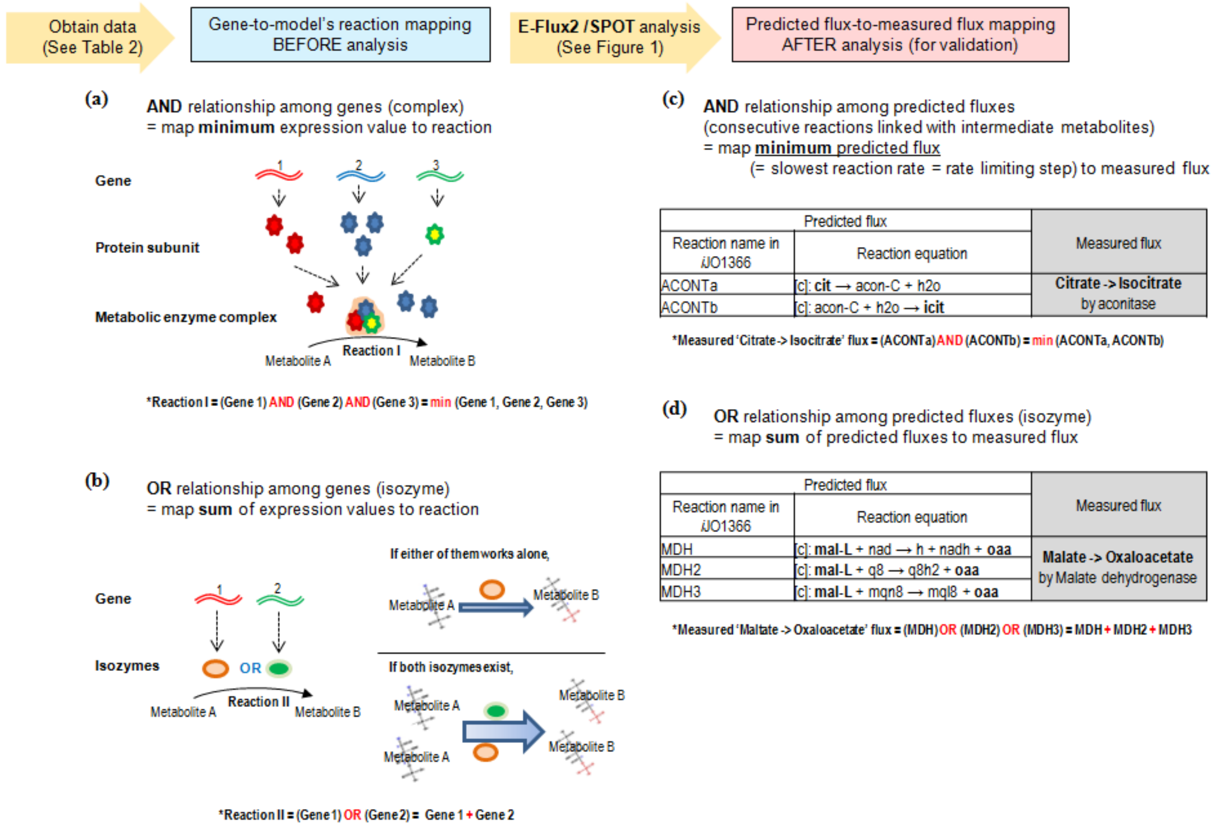

The process for our research can be classified into five steps, which are: 1) obtaining both transcriptomic and fluxomic data measured under the same conditions (See Table 2); 2) mapping gene expression data onto corresponding reactions in the model based on Gene-Protein-Reaction association relationships; 3) creating one of the two template metabolic models depending on carbon source information; 4) solving an optimization problem with one of the two algorithms depending on the availability of information on biomass objective (See Figure 1); and 5) calculating the correlation between predicted and measured fluxes.

Supplementary figure 1a and Supplementary figure 1b illustrate how transcriptomic data were mapped onto corresponding reactions in the model based on Gene-Protein-Reaction association relationships. Supplementary figure 1c and Supplementary figure 1d shows how predicted fluxes were matched with a corresponding measured flux to calculate correlation between them. (a) In the case where an enzyme complex mediates a certain metabolic reaction (AND relationship), we mapped the minimum value of the expression level of the associated genes encoding its subunits to the corresponding reaction since the least-expressed components is likely to determine the final concentration of the complete enzyme complex. (b) If a reaction is catalyzed by isozymes (OR relationship),

we took the sum of the expression values of the associated genes for mapping since the total capacity of the reaction is given by the sum of the capacities of its isozymes. (c) If a certain measured reaction corresponds to the set of consecutive reactions in the model that share intermediate metabolites (AND relationship), the slowest reaction rate also known as the rate-determining step (the minimum flux) among those predicted fluxes was used to match with the corresponding measured flux. (d) In the case where a measured flux corresponds to multiple reactions in the model that mediate an identical chemical conversion independently with each other (OR relationship), the sum of those predicted fluxes was used to match with the corresponding measured flux.

Supplementary figure 2. Rationale for the SPOT method.

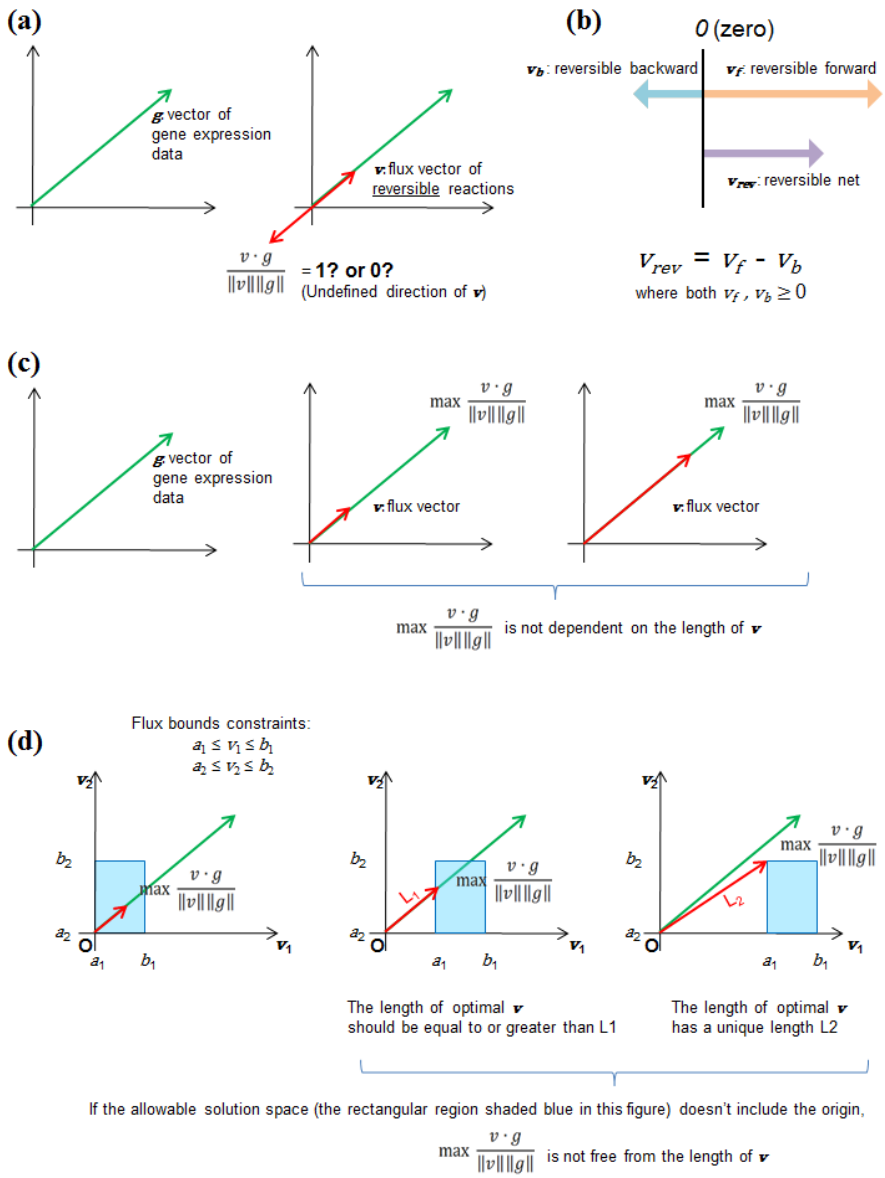

(a) We noticed that the dot product between a flux vector (v, denoted as red arrows in the figure) and its corresponding gene expression data (g, denoted as green arrows in the figure) in the numerator of the objective function cannot be calculated for the set of reversible reactions since the directions of reversible reactions are undefined whereas gene expression data values are always positive. (b) So we decomposed every reversible reaction in the model into two positive irreversible reactions, the forward reaction, v^f, and the backward reaction, v^b, where $v^{rev} = v^f - v^b$, and $v^f, v^b \geq 0$ (c) Usually, the maximum Pearson product-moment correlation is not dependent on the length of v but dependent on the angle between v and g. (d) However, if any of the flux bounds does not

include zero, the origin in the graph, the maximum correlation is no longer independent of the length of the flux vector, v. Thus, it is a prerequisite for using SPOT method to make sure that the allowable solution space includes the origin. The light blue-colored rectangular space shows the allowable solution space that is determined by flux bounds (i.e. a_1, b_1 and a_2, b_2 in the figure) of all reactions (i.e. v_1 and v_2).

Supplementary figure 3. Comparison of the predicted fluxes with the measured fluxes of *S. cerevisiae* data (200mM of acetoin-treated sample of Celton *et al*.).

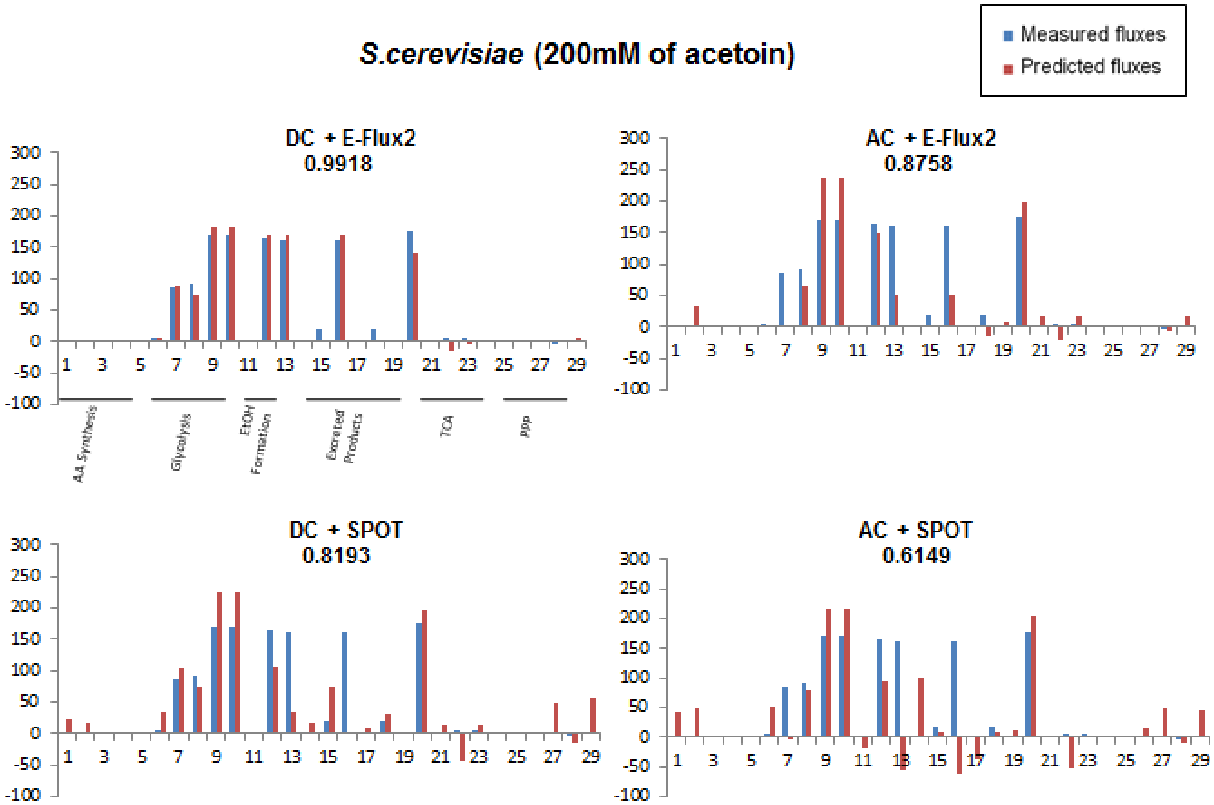

The x-axis represents metabolic reactions used to calculate correlation between the measured (blue bars in the figure) and the predicted fluxes (red bars in the figure), and the y-axis indicates flux value. The scale and the units on the y-axis are based on those of the measured flux.

Supplementary figure 4. Average running time of our algorithm.

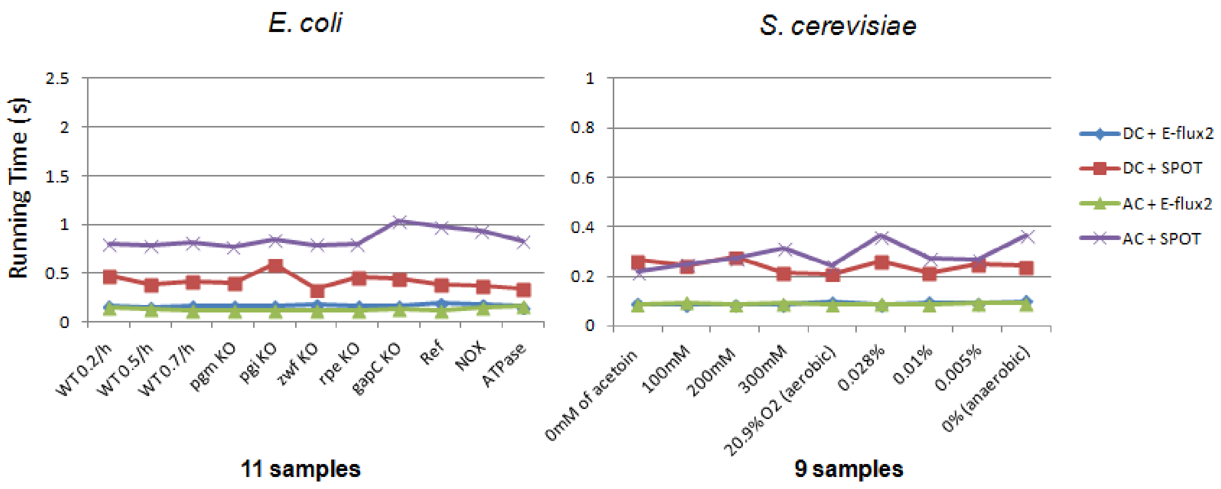

We measured the running time of our algorithm implemented using MATLAB (The Mathworks, Inc., Natick, Mass.) and Gurobi Optimizer (Gurobi Optimization, Inc., Houston, Texas) for all 80 samples (4 simulation methods and 20 samples per simulation method) using the built-in MATLAB function, profile. Regardless of which simulation method is used, our method completes within one second for both *E. coli* and *S. cerevisiae*. Computations were carried out on the Window 8 OS platform using a personal computer with an Intel Core i5 3.10 GHz processor with 8 GB of RAM.

Supplementary figure 5. Exploration of the way to improve a poor performance of Yeast 5 in AC+SPOT.

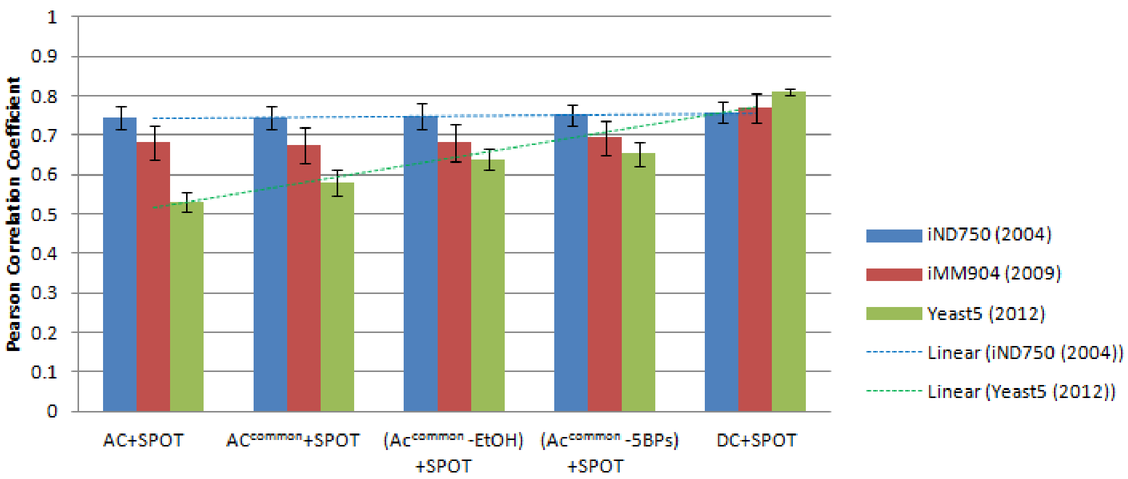

As described in Methods, we built the AC (All possible Carbon sources) model, which has lower bounds of negative infinity for all exchange reactions of possible carbon sources (i.e. external metabolites containing carbon) to simulate the unknown carbon source situation. As listed in Additional file 5, a total of 108, 154, and 158 potential carbon sources were allowed to be taken up by the cell for *i*ND750, *i*MM904, and Yeast 5 models of *S. cerevisiae*, respectively. To test whether Yeast 5 performs worse than the older models because it has more carbon source uptake reactions (leading to more incorrect carbon sources to confound the prediction method), we performed SPOT again after blocking the uptake of model-specific carbon sources, leaving 106 exchange reactions that are common across all three models (see Additional file 7 for details). In other words, we updated the three yeast AC models so that they all have the same set of 106 possible carbon source exchange reactions, which we call the AC^{common} (All possible *common* Carbon sources) model in the figure to distinguish it from the original AC model.

As shown in the AC^{common}+SPOT case of the figure above, although the average correlation of Yeast 5 was improved from 0.5313 to 0.5791 after reducing the number of carbon source uptake reactions to 106, the older yeast models still outperform Yeast 5, which suggests that the lower correlation achieved by SPOT on the Yeast 5 AC model is not simply due to having a greater number of possible carbon sources.

Interestingly, unlike the older models, the performance of Yeast 5 in AC+SPOT was much improved by limiting the uptake of well-known by-products of yeast such as ethanol and glycerol [1]. As shown in the (AC^{common}-

EtOH)+SPOT and (AC^{common}-5BPs)+SPOT case, the correlation of Yeast 5 was further increased from 0.5791 to 0.6397 and 0.6534, when the uptake of ethanol and of well-known five metabolic by-products of yeast (ethanol, carbon dioxide, succinate, glycerol, and acetate) was blocked respectively. The different sensitivity of the performance of each model to changes in the number of carbon sources (see the trend lines in the figure) may indicate a different degree of interconnectivity among intracellular reactions and exchange reactions inherent to the model. Considering that only a small number of preferred carbon sources are consumed by most microorganisms, which is a well-known phenomenon called Caron Catabolite Repression (CCR) [2, 3], reducing the number of possible carbon source uptake reactions by analyzing growth media composition is a sensible way to improve the predictive accuracy of AC+SPOT.

Supplementary Methods

A mathematical justification for dropping the $||\boldsymbol{v}||$ term in the objective function of SPOT

Removing the $||\boldsymbol{v}||$ term in the objective function of SPOT can be justified if a solution of problem 2 (see below, on the right side) also optimizes problem 1 (on the left side):

Problem 1

$$\max \frac{v \cdot g}{||v||}$$

$$\text{subject to} \begin{cases} Av = 0 \\ 0 \leq v \end{cases}$$

$\rightarrow$

Problem 2

$$\max v \cdot g$$

$$\text{subject to} \begin{cases} Av = 0 \\ 0 \leq v \\ ||v|| \leq 1 \end{cases}$$

where v and g has dimension n, and A is a stoichiometric matrix.

Lemma. If v^* optimizes problem 2, then v^* optimizes problem 1.

This lemma can be proved by contradiction.

Let us assume that this statement is false. Then its negation, i.e. if $\boldsymbol{v}^*$ optimizes (i.e. maximizes) problem 2, then $\boldsymbol{v}^*$ does not optimize problem 1, should be true. In other words, we assume that another vector $\boldsymbol{v}$ exists such that

$$\frac{v \cdot g}{||v||} > \frac{v^* \cdot g}{||v^*||}$$

where $v \neq v^*$.

Let $v' = \frac{v}{||v||}$, then $||v'|| = \frac{1}{||v||} \times ||v|| = 1$.

Substituting v' into the term on the left side of the inequality above gives $v' \cdot g$.

In addition, since v^* optimizes problem 2 by assumption, $\|v^*\| \leq 1$. Note that no optimal solution to problem 2 has $\|v\| < 1$ because g is a non-negative vector and we assume that $g \neq 0$ (for otherwise, the problem is trivial). Thus, if $\|v\| < 1$, the objective of problem 2 can always be increased by scaling up v until $\|v\| = 1$. Hence $\|v^*\| = 1$.

The right side of the inequality, thus, is equal to $v^* \cdot g$. Therefore,

$$v' \cdot g > v^* \cdot g$$

where $\|v'\| = 1$.

This contradicts our assumption that v^* is a vector that optimizes problem 2. Since the negation is impossible (false), the original statement is true. Note that the lemma is still valid if the number used to limit $\|v\|$ in problem 2 is any constant $c > 0$.

A mathematical proof of the uniqueness of SPOT solutions

SPOT (equation (8) in the main manuscript) is defined as the following optimization problem:

$$\max f'v$$

$$\text{subject to} \begin{cases} Av = 0 \\ 0 \leq v \\ \|v\| \leq 1 \end{cases}$$

where v has dimension n, $f \geq 0$ and A is a stoichiometric matrix.

Lemma. If the optimal value of the SPOT problem is strictly positive, then its solution is unique.

Proof. *Step 1.* First we prove that $\|v\| = 1$. If $\|v\| < 1$ then for ϵ sufficiently small $\omega = v + \epsilon \frac{v}{\|v\|}$ satisfies $\|\omega\| < 1$ (and the other constraints) and

$$f^{'}\omega = f^{'}v\left(1 + \frac{\epsilon}{\|v\|}\right) > f'v$$

because $f^{'}v > 0$, contradicting the maximality of $f'v$.

Step 2. Because of the maximality of $f'v$, the affine plane $\{\omega : f^{'}\omega = f'v\}$ is a supporting plane for the convex set $C = \{\omega : \omega \in ker(A), \omega \geq 0, \|\omega\| \leq 1\}$. Moreover, by Step 1, such a plane cannot be orthogonal to any linear space containing the vector v. We conclude that the affine plane intersects C only at v, thus v is the only point of the maximum for the linear functional $\omega \rightarrow f'\omega$.

Remark. Notice that the condition of strict positivity of the maximum if necessary. Indeed if $ker(A)$ is contained on a coordinate plane, say the first: $ker(A) \subset \{\omega : \omega_1 = 0\}$, then the vector $f = e_1$ satisfies $f^{'}\omega = 0$ for every vector in $ker(A)$ and there is no uniqueness.

Calculation of the possible range of correlation between the measured fluxes and the predicted fluxes

For standard FBA and E-Flux, the methods that do not give a unique metabolic flux distribution, the possible range of correlation between the measured fluxes and the predicted fluxes were calculated. Given information on the measured fluxes, the minimum and the maximum correlations of standard FBA can be calculated using the following two steps of optimization:

Step 1. standard FBA → Step 2. calculation of the possible range of correlation

$$z^* = \max f'v \quad \rightarrow \quad \text{min/max} \frac{v_p \cdot v_m}{\|v_p\|\|v_m\|}$$

$$\text{subject to} \begin{cases} Sv = 0 \\ a_j \le v_j \le b_j \end{cases} \qquad \text{subject to} \begin{cases} Sv = 0 \\ a_j \le v_j \le b_j \\ f'v = z^* \end{cases}$$

where v is a flux vector representing the reaction rates of the n reactions in the network, f is a coefficient vector defining the organism's objective function, S is the stoichiometric matrix, and a_j and b_j are the minimum and maximum reaction rates through reaction j. The vectors v_p and v_m shown in the objective function of Step 2 are the predicted and measured vectors of intracellular fluxes, respectively, and $\|\cdot\|$ denotes the l^2 norm.

The average correlation of standard FBA in Table 3 was calculated using solutions obtained in Step 1 under our computational settings (see Methods in the main manuscript for the detailed settings). Step 2 allows us to find a metabolic flux distribution which achieves theoretically maximal or minimal correlation with the measured fluxes while maintaining the optimal biomass flux, denoted as z^* here. The nonlinear optimization problem in Step 2 was solved using the sequential quadratic programming (SQP) algorithm provided by the MATLAB function fmincon.

Importantly, the maximum possible correlation can be calculated only when we already have the known measured flux datasets. There is no way to force each method to produce a metabolic flux distribution which achieves the best correlation with the measured fluxes. Our methods were developed during the process of finding that way and of rigorously testing various strategies.

In the same way, the lower and upper bound of correlations of E-Flux can be calculated as follows:

Step 1. E-Flux

$$z^* = \max f'v$$

$$\text{subject to} \begin{cases} Sv = 0 \\ a_j^e \le v_j \le b_j^e \end{cases}$$

$\rightarrow$

Step 2. calculation of the possible range of correlation

$$\text{min/max } \frac{v_p \cdot v_m}{\|v_p\| \|v_m\|}$$

$$\text{subject to} \begin{cases} Sv = 0 \\ a_j^e \le v_j \le b_j^e \\ f'v = z^* \end{cases}$$

where, for all j,

$$a_j^e = \begin{cases} -g_j, \text{if } a_j < 0, \\ 0, \text{if } a_j \ge 0, \end{cases} \text{ and } b_j^e = \begin{cases} g_j, \text{if } b_j > 0, \\ 0, \text{if } a_j \le 0. \end{cases}$$

References

1. Nevoigt E: **Progress in metabolic engineering of Saccharomyces cerevisiae.** *Microbiol Mol Biol Rev* 2008, **72**:379–412.

2. Görke B, Stülke J: **Carbon catabolite repression in bacteria: many ways to make the most out of nutrients.** *Nat Rev Microbiol* 2008, **6**:613–624.

3. Vinuselvi P, Kim MK, Lee SK, Ghim CM: **Rewiring carbon catabolite repression for microbial cell factory**. *BMB Rep* 2012:59–70.
