# Supplementary material for: E-Flux2 and SPOT: Validated Methods for Inferring Intracellular Metabolic Flux Distributions from Transcriptomic Data
Source: PLoS One. 2016 Jun 21;11(6):e0157101. doi: 10.1371/journal.pone.0157101 (PMC4915706; doi:10.1371/journal.pone.0157101)

INTRODUCTION

------------

A general tutorial on how to run E-Flux2 and SPOT in MOST can be found at <http://most.ccib.rutgers.edu/tutorials.html>. This document covers more specific and detailed information on how to reproduce our results using the transcriptomic data and model files that are provided as Supplementary data.

REQUIREMENTS

------------

**1. MOST**

The installation guide for MOST can be found at <http://most.ccib.rutgers.edu/installing_most.html>.

**2.[recommended] Gurobi solver** (version 5.6.3 or better, free academic)

Gurobi is an efficient and versatile solver that can be used for both E-Flux2 and SPOT. It is freely available for academic users, which is downloadable at <http://www.gurobi.com/downloads/download-center>. Once it is installed, Gurobi can be set as a main solver of MOST as shown in the picture below. Please see <http://most.ccib.rutgers.edu/select_solvers.html> for details on how to select solvers.


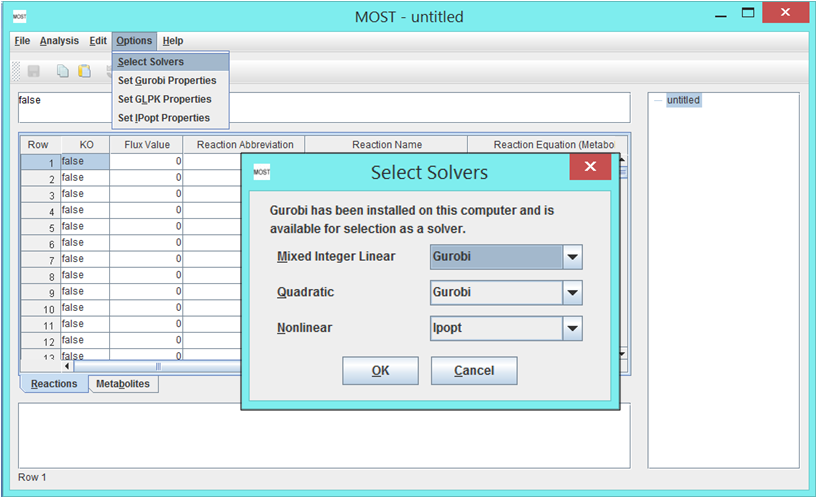


**3. Transcriptomic data and model files** (can be found in Additional Files 3 and 4 of this paper)

RUNNING **E-Flux2** via MOST

------------------------

**Step 1. Load Model**

To load an SBML model in Additional File 3, go to File, then click Load SBML. If necessary, the model can be edited (e.g. changing the objective function) after being loaded depending on your experiment. The E. coli iJO1366 model will be used for this tutorial.


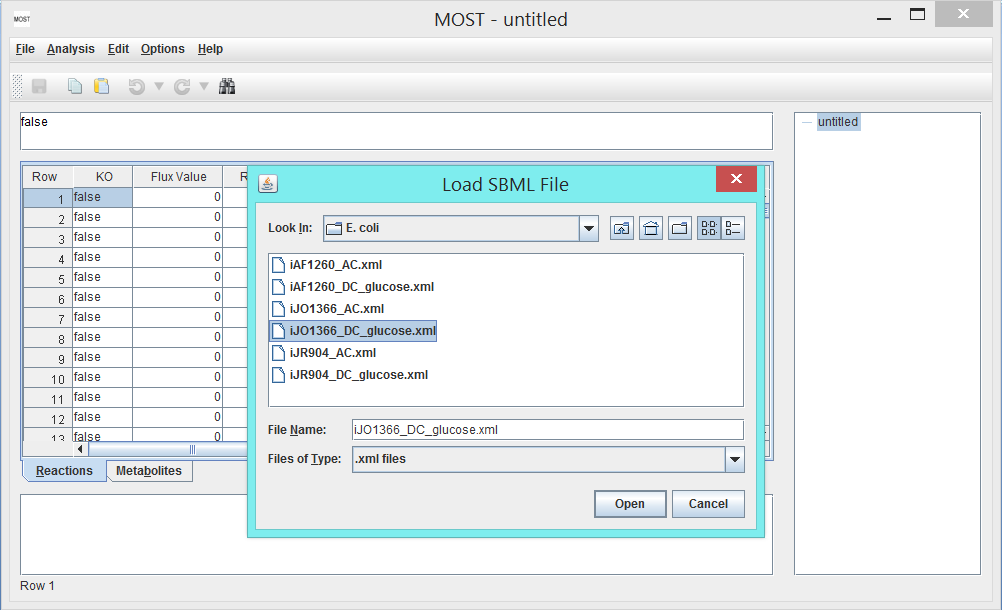


**Step 2. Set biological objective**

As described in the main manuscript, E-Flux2 can be used if a suitable objective is known. In this study, biomass flux was chosen as the biological objective to be maximized. An easy way to find the biomass reaction is to use Find/Replace dialog using Ctrl-F keystroke. Once this reaction is located, scroll right to the Biological Objective column (the 10th column) and set it to 1 as shown below. Since all models in Additional File 3 already have the biomass reaction objective set to 1, this will not need to be changed at this moment.


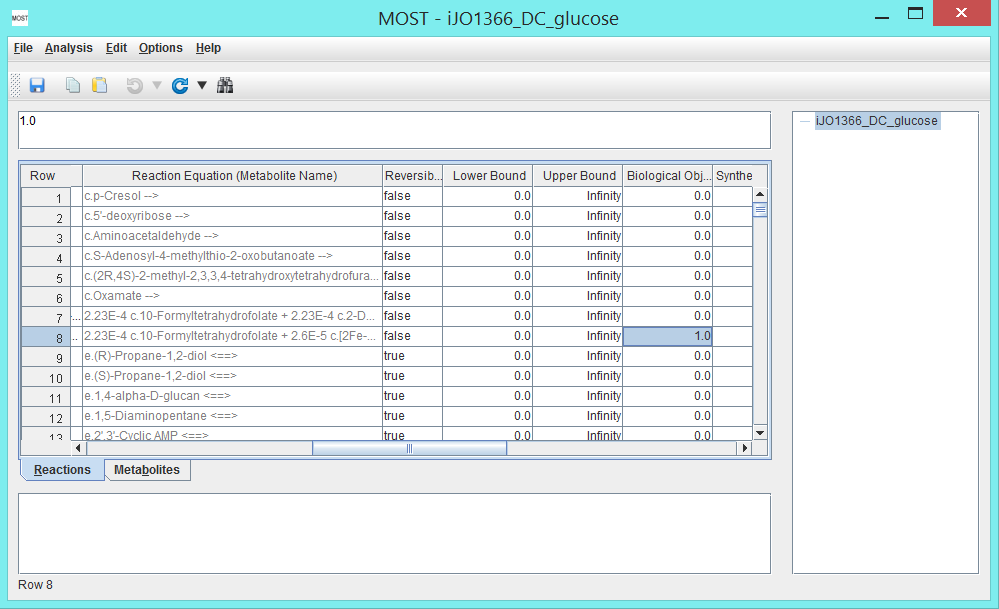


**Step 3. Load gene expression file**

E-Flux2 requires a CSV file with two columns where the first column is the gene names and the second column is the gene expression of one experimental condition as shown below:


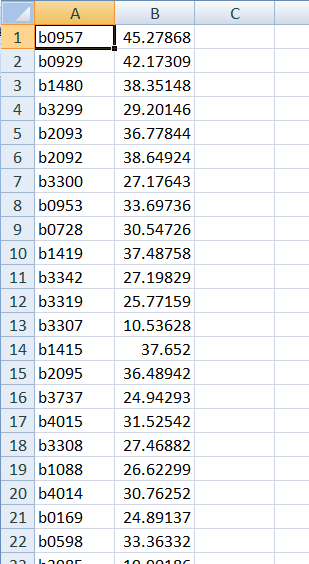


Since Additional File 4 includes transcriptomic data of 20 different experimental conditions (11 in *E. coli* and 9 in *S. cerevisiae*), creating a new CSV file will be necessary by copying two columns (one for the gene names, the other for the gene expression levels) from Additional File 4 and pasting them to a new file as follows:


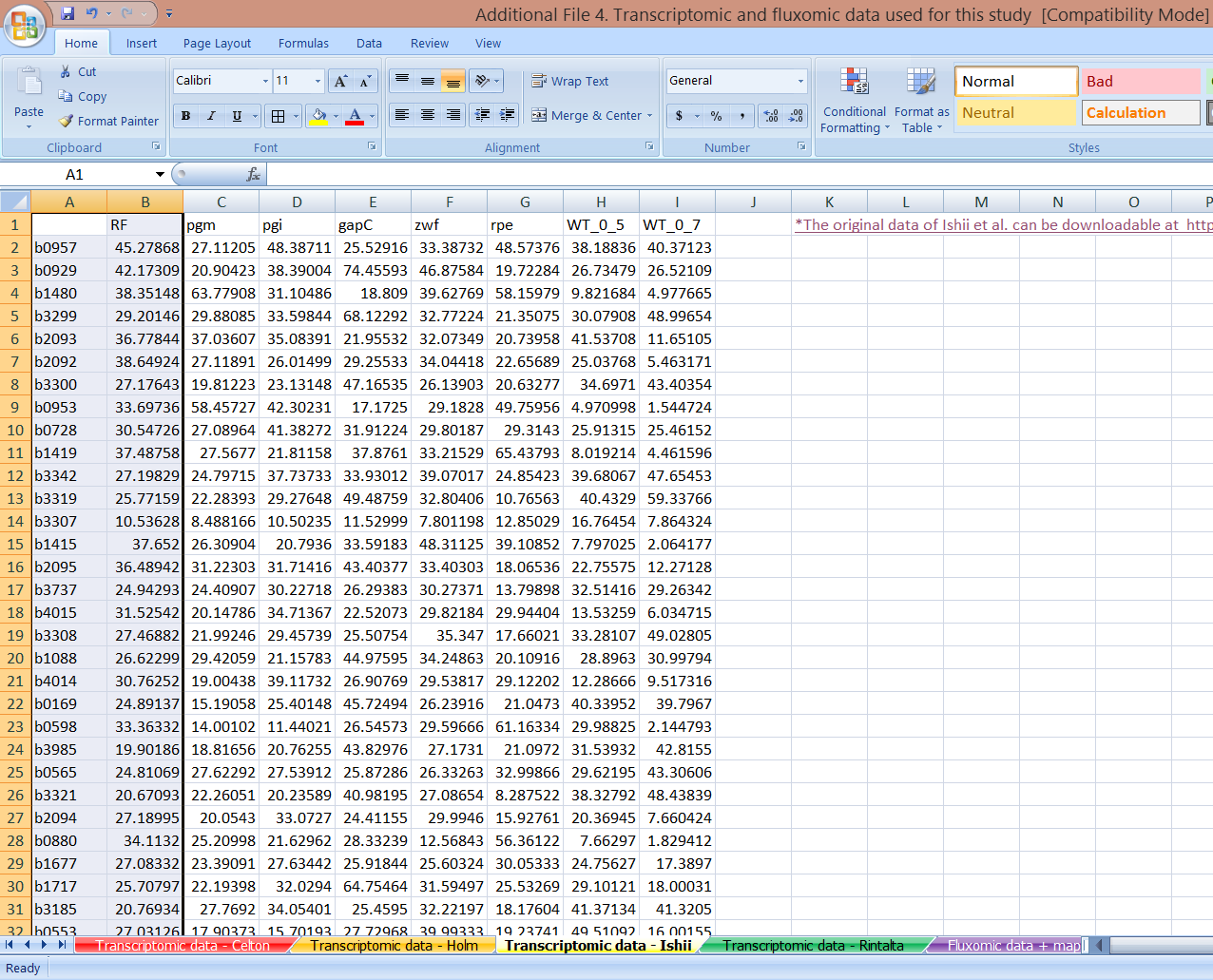


**Step 4. Run Analysis**

Select Analysis → E-Flux2 (Alt+A then Alt+E).A file browser will appear. Select the gene expression csv created in Step 3, then click the Open button on the file browser:


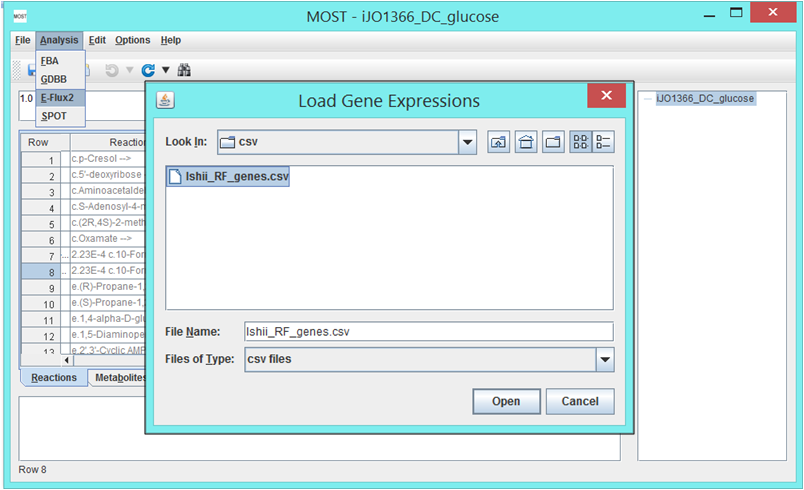


After E-Flux2 is finished, a new link of the optimization result will be added to the tree on the upper right panel (See the picture below), and results will be displayed in the console. The numerical values in the Flux Value column shows the predicted metabolic fluxes.


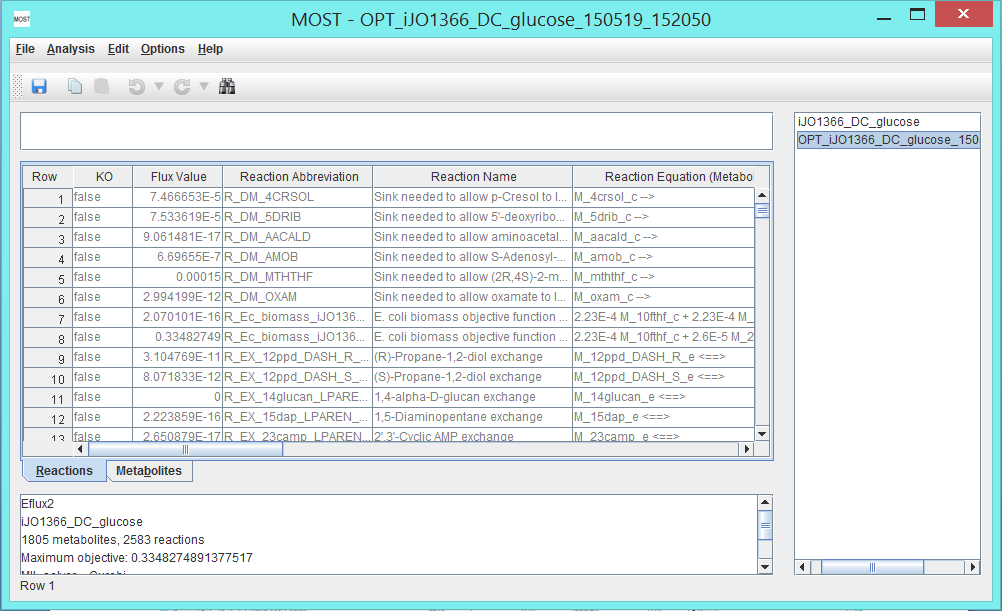


RUNNING **SPOT** via MOST

---------------------

If a suitable objective is unknown, SPOT can be used.

**Step 1. Load Model**

To load an SBML model in Additional File 3, go to File, then click Load SBML. If necessary, the model can be edited (e.g. changing the number of possible carbon source uptake reactions) after being loaded depending on your experiment. The E. coli iJO1366 model will be used for this tutorial.


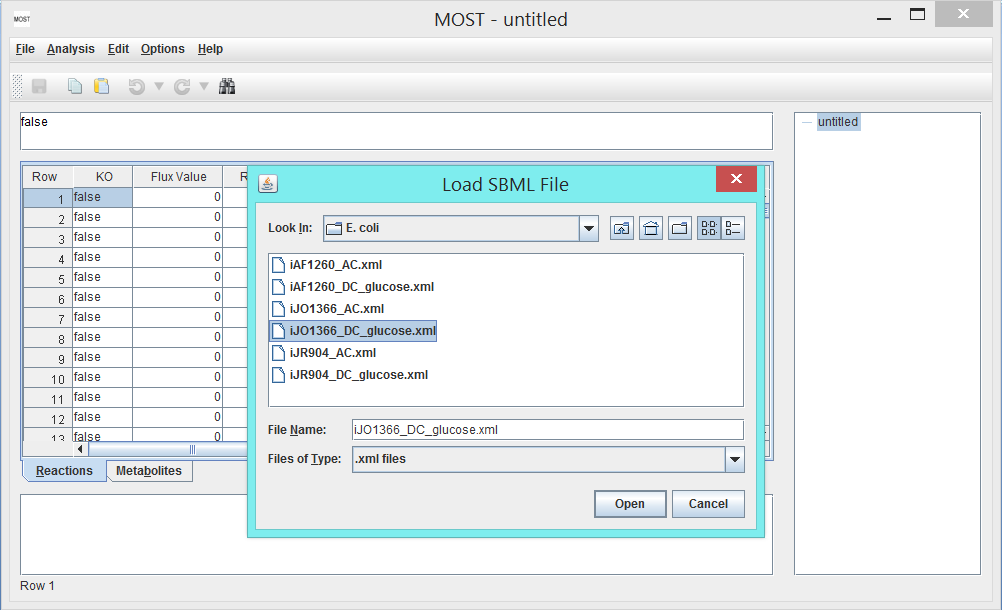


**Note: SPOT does not require a Biological Objective to be set.**

Although the models in Additional File 3 have the biomass reaction objective set to 1, this will not need to be changed at this moment since SPOT does not use information in the Biological Objective column to run.

**Step 2. Load gene expression file**

SPOT requires a CSV file with two columns where the first column in the gene name and the second column is the gene expression of one experimental condition as shown below:


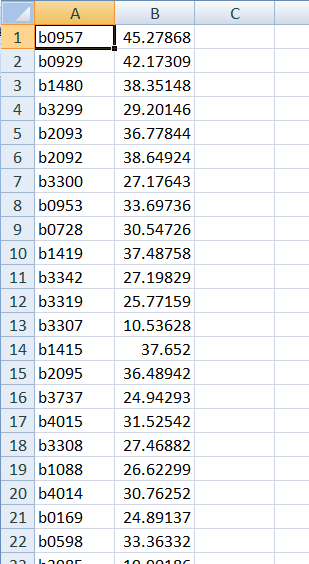


Since Additional File 4 includes transcriptomic data of 20 different experimental conditions (11 in *E. coli* and 9 in *S. cerevisiae*), creating a new CSV file will be necessary by copying two columns (one for the gene names, the other for the gene expression levels) from Additional File 4 and pasting them to a new file as follows:


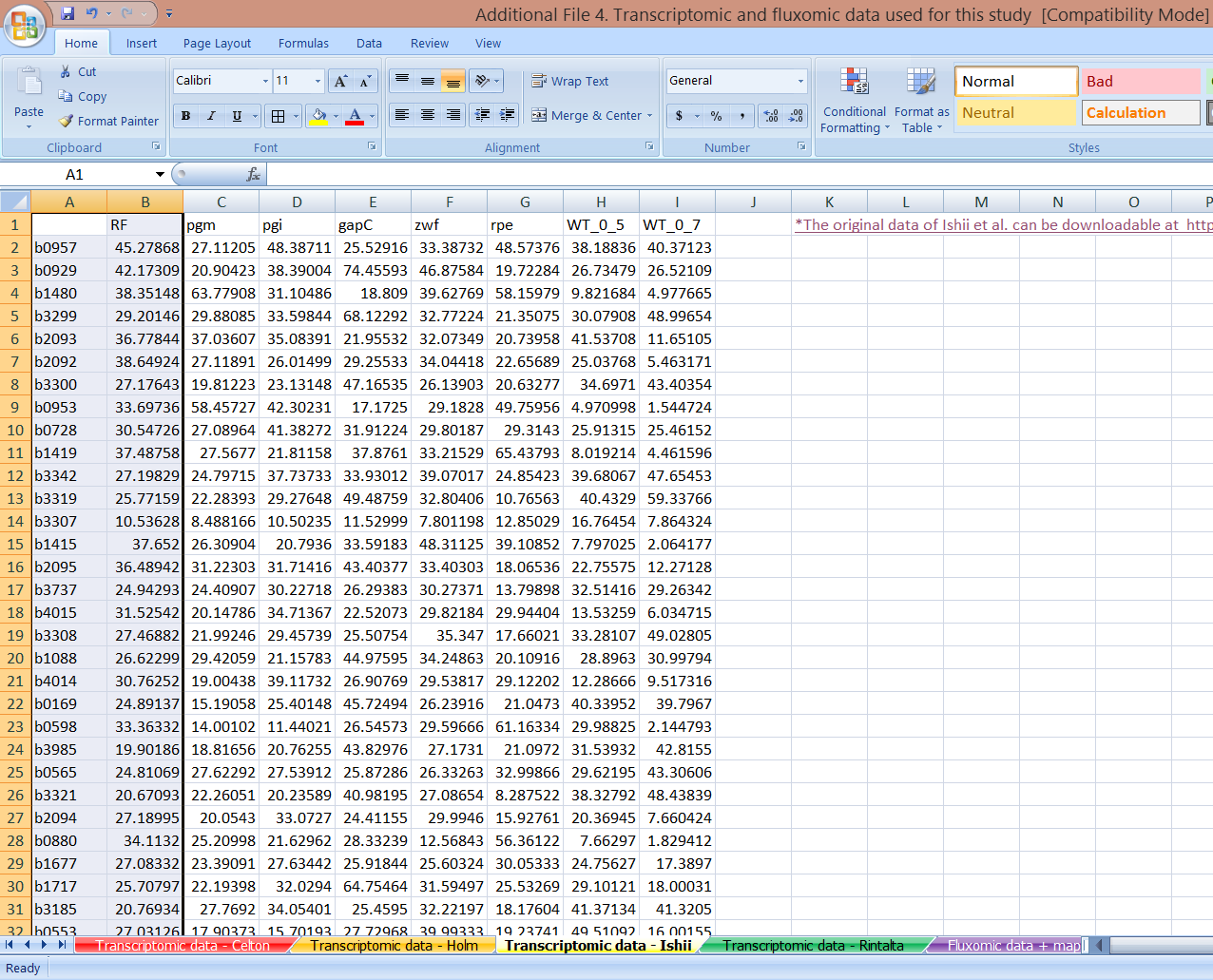


**Step 3. Run Analysis**

Select Analysis → SPOT (Alt+A then Alt+S).A file browser will appear. Select the gene expression csv created in Step 2, then click the Open button on the file browser:


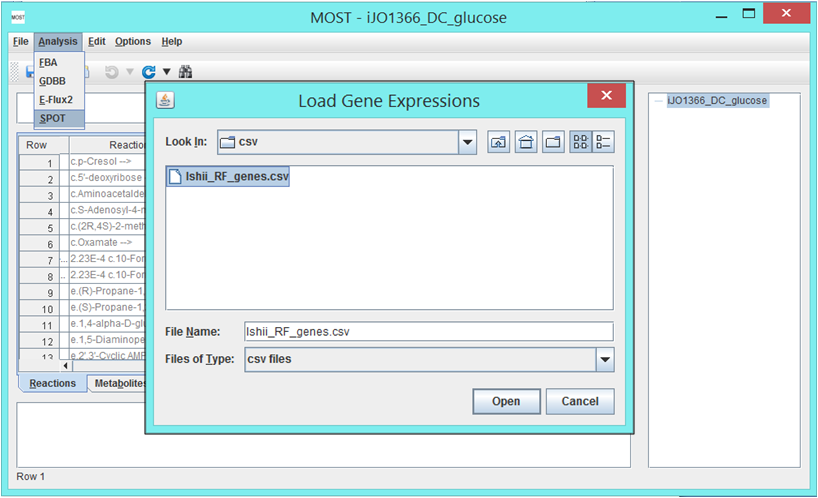


After SPOT is finished, a new link of the optimization result will be added to the tree on the upper right panel (See the picture below), and results will be displayed in the console. The numerical values in the Flux Value column shows the predicted metabolic fluxes.


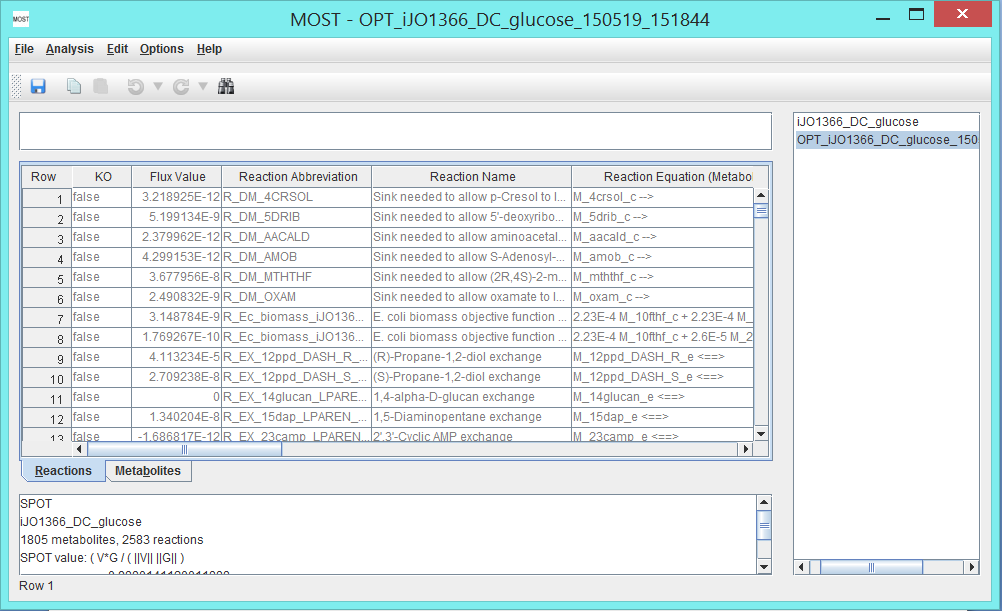

Supplement: S2 File — E-Flux2 and SPOT are implemented in the open source package MOST (http://most.ccib.rutgers.edu/). This document covers information on how to reproduce our results in MOST using the model files and transcriptomic data that are provided as Datasets S1 and S2. (DOC) [file pone.0157101.s005.doc]
