# Supplementary material for: E-Flux2 and SPOT: Validated Methods for Inferring Intracellular Metabolic Flux Distributions from Transcriptomic Data
Source: PLoS One. 2016 Jun 21;11(6):e0157101. doi: 10.1371/journal.pone.0157101 (PMC4915706; doi:10.1371/journal.pone.0157101)
Supplement: S3 Dataset — Instructions on using it with the dataset of Ishii et al. can be found in README.doc. The source code written in Java is also available (see S2 File for details). This MATLAB code includes calculation of the Pearson correlation with measured flux data. (ZIP) [file pone.0157101.s003.zip › README.doc]

INTRODUCTION

------------

This document covers information on how to run E-Flux2 and SPOT in MATLAB using the code, model, and data files that are provided as supplementary materials (Additional files 8, 3, and 4, respectively). The source code written in Java is also available, which is open to the public at [http://most.ccib.rutgers.edu](http://most.ccib.rutgers.edu/) (see Addtional file 2 for details).

REQUIREMENTS

------------

**1. SBMLToolbox**

SBMLToolbox is necessary to convert an SBML model into a MATLAB data structure (Keating et al., *Bioinformatics*, 2006). The installation guide for SBMLToolbox can be found at <http://sbml.org/Software/SBMLToolbox>.

**2. Gurobi solver** (version 5.6.3 or better, free academic)

Gurobi is an efficient and versatile solver that can be used for both E-Flux2 and SPOT. It is freely available for academic users, which is downloadable at <http://www.gurobi.com/downloads/download-center>.

**3. Transcriptomic data and model files** (can be found in Additional Files 3 and 4 of this paper)

PROCEDURES

------------------------

**Step 1. Convert an SBML to a MATLAB data structure (by running sbml_to_mat.m)**

Many genome-scale metabolic models are provided in SBML format. To use an SBML model in the MATLAB environment, it is necessary to convert it into a MATLAB data structure. To do so, run **sbml_to_mat.m**. Copy an SBML model in Additional File 3 (The E. coli iJO1366 Determined Carbon source model will be used for this tutorial) to the Current Folder where sbml_to_mat.m is located, then enter 'sbml_to_mat(the name of the model file)' at the MATLAB command line as follows:

The function returns the SBML model's corresponding MATLAB data structure called fbamodel.

Save the workspace variable, fbamodel, to MAT-file for further use.

**Step 2. Convert gene expression data to MAT-file (by running expr_txt_to_mat.m)**

To use the transcriptomic data in Additional file 4, it is necessary to convert it into MAT-file as well. The MATLAB script named **expr_txt_to_mat.m** shows how this process can be done. Copy data in the worksheet of interest (the dataset from Ishii et al. will be used for this tutorial) from Additional File 4 and paste them to a new file. Then, save the file as a Tab-delimited text file as shown below:

```
Note that the first column of gene expression data should have gene symbols/names used in the GPR association of the network so that the data can be integrated to the model.
 Move the saved text file (e.g. test_expr.txt) to the Current Folder where expr_txt_to_mat.m is run from, then enter 'expr_txt_to_mat' the at the MATLAB command line as follows:
```

expr_txt_to_mat.m reads the text file (e.g. test_expr.txt) and returns the corresponding MAT-file (e.g. test_expr.mat) that can be used for analysis in the MATLAB environment. The script can be modified depending on the input file such as the number of columns it has.

**Step 3. Prepare measured flux data files**

In this study, measured fluxes were used to validate the predictive accuracy of our method by calculating the uncentered Pearson correlation with predicted fluxes. Since some of the measured fluxes are not directly matched with predicted fluxes of the model in a 1-to-1 relationship, we need a 1) matching table that shows this relationship. The matching tables can be found on the fifth worksheet of Additional file 4. Select one depending on the model and dataset that will be used for analysis, then save it as a Tab-delimited text file (e.g. fluxmatched.txt for this tutorial) as follows:

2) On the same worksheet, select measured flux values in the condition of interest, then save them as a Tab-delimited text file, too (e.g. measured_flux_rf.txt for this tutorial) as the following:

**Step 4. Perform Analysis (by running eflux2.m or spot.m)**

***E-Flux2**

As described in the main manuscript, E-Flux2 can be used if a suitable objective is known. In this study, biomass flux was chosen as the biological objective to be maximized. This information can be found from one of the fields of fbamodel named *f*, which is a coefficient vector where the element defining the organism’s objective function is set to 1 (see below).

Run E-Flux2 by entering '[eflux2_flux, correl_m] = **eflux2**(a number indicating the experimental condition of interest under which both transcriptomic data and fluxtomic data were measured)' at the MATLAB command line as shown in the picture below. For this tutorial, the dataset from Ishii et al. was used. It consists of 8 experimental conditions, and 1 indicates Reference (WT 0.2 h^-1^) condition.

After E-Flux2 is finished, two variables called correl_m and eflux2_flux for the correlation and for the predicted metabolic fluxes, respectively, will be added to the Workspace (See the picture above). Numerical flux values can be examined in more detail by double-clicking eflux2_flux.

***SPOT**

If a suitable objective is unknown, SPOT can be used.

Run SPOT by entering '[spot_flux, correl_m] = **spot**(a number indicating the experimental condition of interest under which both transcriptomic data and fluxtomic data were measured)' at the MATLAB command line as shown in the picture below. For this tutorial, the dataset from Ishii et al. was used. It consists of 8 experimental conditions, and 1 indicates Reference (WT 0.2 h^-1^) condition.

After SPOT is finished, two variables called correl_m and spot_flux for the correlation and for the predicted metabolic fluxes, respectively, will be added to the Workspace (See the picture above). Numerical flux values can be examined in more detail by double-clicking eflux2_flux.
